# Research on Spatial Information Network Vulnerability Analysis Methodology Based on Multi-Layer Hypernetworks

**DOI:** 10.3390/s26051570

**Published:** 2026-03-02

**Authors:** Xiaolan Yu, Wei Xiong, Yali Liu

**Affiliations:** Graduate School, Space Engineering University, Beijing 101416, China; yuxiaolan@hgd.edu.cn (X.Y.);

**Keywords:** spatial information network, multi-layer hypernetwork, vulnerability, hypernetwork overlap

## Abstract

**Highlights:**

This paper mainly adopts a multi-layer hypernetwork approach to establish a spatial information network model, proposes computational formulas for communication, navigation, and remote sensing task efficiency, implements vulnerability analysis under spatial information network multi-task scenarios, and proposes a hypernetwork overlap removal strategy and hardening strategy that can reliably and comprehensively reduce the vulnerability of spatial information networks.

**What are the main findings?**

- A calculation formula for communication, navigation, and remote sensing task efficiency is proposed.
- A node mining method with hypernetwork overlap is proposed.

**What are the implications of the main findings?**

- Establishing a spatial information network vulnerability analysis model in multitasking scenarios.
- Achieving a decrease in spatial information network vulnerability in multitasking scenarios.

**Abstract:**

As the core infrastructure for providing all-weather, full-coverage, high-speed, and diversified information services, spatial information networks (SINs) possess significant social, economic, and military value. However, due to the inherent characteristics of their network architecture, SINs are susceptible to core service paralysis and functional failure under large-scale targeted attacks or random disturbances, posing a critical bottleneck that constrains their stable operation. Current research on SIN vulnerability is predominantly confined to a single network topology perspective, lacking an integrated consideration of the task execution perspective. Consequently, it fails to accommodate the dual requirements of “network topology stability” and “task execution effectiveness”. To address the aforementioned research needs and challenges, this study adopts a “topology-task” dual-perspective fusion approach and proposes a vulnerability analysis framework for SINs that integrates multi-layer networks and hypernetworks. First, a two-layer SIN topology model encompassing the user layer and the satellite layer is constructed. Leveraging hypernetwork theory, information tasks involving multiple network entities are formally defined, and an integrated multi-layer hypernetwork model is established. Second, based on distinct task types, three categories of task efficiency evaluation metrics are defined, and corresponding quantitative methods for calculating SIN vulnerability are derived. Third, during the vulnerability analysis phase, a novel strategy for identifying and removing overlapping nodes in hypernetworks is introduced to enable precise localization of critical nodes within the network. Concurrently, a pre-attack node hardening strategy is designed to minimize the impact of attacks on network performance. Finally, through systematic analysis of vulnerability performance and critical node characteristics under different node removal strategies, the results demonstrate enhanced network performance. The effectiveness of the proposed method is validated by comparing the defense performance of the hardening strategy across various attack scenarios. To verify the feasibility and superiority of the proposed method, this study designs 5 × 5 groups of simulation experiments with varying network parameters. The results indicate that, compared with traditional methods, the proposed strategy can more accurately identify core nodes affecting the stable operation of SINs, significantly reducing network vulnerability and improving network survivability. In addition, a comprehensive sensitivity analysis of SIN vulnerability is conducted from three key influencing dimensions—mission scale, satellite count, and constellation configuration—clarifying the impact of each dimension on network invulnerability. Thus, this paper provides a reliable theoretical foundation and technical support for the planning, design, optimal deployment, and operation and maintenance management of SINs.

## 1. Introduction

The spatial information network (SIN) is a comprehensive system for high-speed information transmission and processing, encompassing satellites, near-space platforms, and ground stations as nodes. It integrates three-layer links spanning space, air, and ground, and is characterized by node heterogeneity, dynamic topological changes, and cross-domain spatiotemporal coupling. As a national-level strategic infrastructure, the SIN supports critical missions such as broadband access in remote regions, military command and control, and emergency rescue operations. Currently, low-orbit mega-constellations such as Starlink and OneWeb have deployed thousands of satellites to provide low-latency broadband services. Meanwhile, various countries are advancing the development of “space-based comprehensive information networks” [1], forming a multi-layered architecture that integrates low Earth orbit (LEO), medium Earth orbit (MEO), and geostationary Earth orbit (GEO) segments. As the number of satellites, the scale of ground users, and the diversity of payloads continue to grow, these developments not only drive the evolution of SINs through increasing demands but also pose significant challenges to the performance and security of information services.

In recent years, scholars worldwide have conducted extensive research on the structural characteristics of spatial information networks (SINs), with a primary focus on topological vulnerability analysis and corresponding hardening strategies. Existing analytical approaches can be broadly categorized into three types: traditional graph theory-based network topology analysis [2,3]; machine learning-based methods that leverage data-driven predictive capabilities [4,5]; and task-oriented approaches that incorporate high-level network task mapping [6,7]. Among these, machine learning methods (e.g., [4,5]) utilize historical data to predict network states or attack patterns, demonstrating strong adaptability and predictive accuracy when sufficient data are available. However, in the context of emerging SIN architectures lacking extensive historical data on large-scale attacks, model training and generalization remain challenging. In contrast, task-oriented methods align more closely with operational requirements and represent a prominent direction in current frontier research. Systematic vulnerability analysis of SINs is therefore essential to ensure the robustness of their core functionalities.

Research on the vulnerability of spatial information networks (SINs) is currently progressing along several key dimensions. The first dimension involves the analysis of critical nodes and topological structures. Early studies concentrated on identifying key nodes within static topologies, proposing metrics such as node strength and degree centrality based on graph models to assess weak links in satellite networks [8]. Subsequently, researchers recognized that SIN topologies evolve periodically with satellite orbits and introduced time-slice-based dynamic modeling methods, which partition the network over a given period into multiple static subgraphs to capture the time-varying characteristics of node importance [9]. Building on this foundation, an optimized supra-adjacency matrix (OSAM) modeling approach was proposed, which improves intra-layer relationship matrices by incorporating edge weights during the construction of the optimized super adjacency matrix [10]. Although these studies have conducted in-depth exploratory analyses of both static and dynamic network structures, they do not integrate network topology with functional implementation, resulting in a lack of application-level insight.

To further investigate the coupling between functional requirements and network structures, vulnerability analysis for single-layer networks [11] can only capture the impact of physical entity attacks. However, SINs are interdependent networks composed of multiple coupled subnets—such as communication, navigation, and early warning layers. Research grounded in interdependent network theory has begun to explore the cascading effects of cross-layer coupling failures. In SINs, entities not only transmit data and exchange information through nodes and links but also collaborate logically to form higher-level information service networks—such as navigation services—via top-layer protocols. Within these logical networks, entities may not have direct physical connections but still cooperate through multiple relationships. In complex networks, nodes represent entities and edges denote binary relationships between them; consequently, complex networks are limited in their ability to represent multiple logical relationships among entities. To address this, the literature [12] proposed a hybrid method combining complex networks and hypergraphs for SIN analysis, integrating information services with network topologies to establish a resilience assessment framework based on hardening–disruption–recovery strategies.

Building upon the aforementioned analysis, it is evident that hypergraphs can effectively represent multiple relationships within systems, thereby enhancing modeling accuracy, while value-weighted approaches can adequately capture variations in task requirements. Accordingly, we integrate complex network and hypernetwork methodologies, focusing on deductive analysis that bridges the intrinsic relationship between network structure and mission requirements. This complements data-driven methods and offers a novel approach for assessing inherent network vulnerability in the absence of prior attack samples. Specifically, we propose a multi-layer hypernetwork model for SINs that encompasses both the ground node layer and the satellite network layer, addressing the vulnerability analysis problem arising from the coupling between uneven user demands and structurally ordered satellite networks during the practical deployment and application of SINs. Within this framework, more appropriate information service quantification criteria are defined based on specific task requirements, and vulnerability is examined across two phases: node removal strategies and hardening strategies. The objective is to analyze SIN vulnerability under different removal strategies, thereby providing administrators and network planners with new analytical perspectives for the management and design of network topologies. In summary, the main contributions of this paper are as follows:

First, overcoming the limitation of traditional models that separate topology from demand, we construct a multi-layer hypernetwork SIN model based on complex network and hypernetwork theories. By integrating satellite–terrestrial links and establishing a bidirectional “topology–demand” mapping, this work provides a novel modeling framework for the topological analysis of SINs.

Second, addressing the homogenization drawback of conventional network efficiency metrics, we propose three types of task-specific efficiency indicators tailored to the integrated demands of communication, navigation, and remote sensing. These indicators solve the long-standing challenge of accurately describing the diverse capabilities of SINs and offer new evaluation metrics for assessing SIN performance.

Third, to overcome the limitation of node identification that is detached from mission context, we introduce an identification method based on hypernetwork overlap degree, which prioritizes critical nodes in close conjunction with task requirements. This approach provides a new analytical perspective for vulnerability prevention and control in SINs.

The remainder of this paper is organized as follows: Section 2 analyzes the composition of SINs and identifies the key problems and challenges they face. Section 3 introduces the fundamental theories of hypernetworks, details the construction process of the multi-layer hypernetwork model for SINs, and presents the methodological principles and quantitative indicators used for vulnerability analysis. Section 4 conducts experimental validation and discusses the corresponding results. Section 5 examines the relationship between SIN vulnerability and factors such as satellite count and task scale. Finally, the paper concludes with a summary of the main findings and contributions.

## 2. Composition and Problem Analysis of Spatial Information Network

The primary components of SINs consist of ground-level user nodes and space-based satellite networks. Structurally, SINs exhibit two key characteristics. On one hand, the satellite network layer comprises a large number of nodes, and its topology exhibits periodic and highly dynamic features; moreover, satellite payload failures are difficult to repair. Therefore, to ensure the core functionality of SINs, the topology of the satellite network must be designed to possess strong resilience against inevitable failures. On the other hand, the ground-level user layer is characterized by an uneven global distribution, with significant disparities in user demand between developed and underdeveloped regions. This contrasts sharply with the relatively uniform distribution pattern of the satellite network layer. Consequently, during the practical ground-level application of SINs, a coupling effect emerges between unbalanced user demands and structurally ordered satellite networks. Vulnerability is thus not merely an inherent property of the network but also a result of dynamic interactions with specific user distribution patterns. To achieve a more comprehensive and accurate vulnerability analysis of SINs, this section systematically examines both the satellite network layer and the ground-level user layer, clarifying the current state and challenges associated with SIN vulnerability analysis.

### 2.1. Composition of Spatial Information Networks

**Figure 1:**
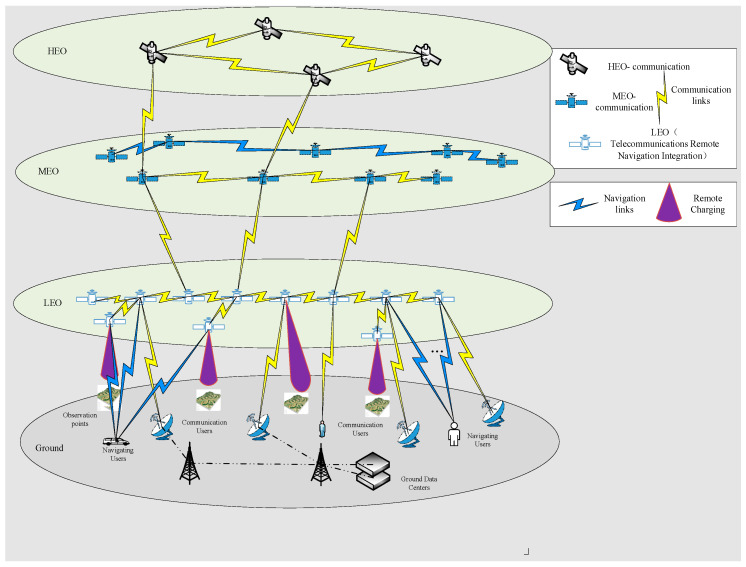
Composition of spatial information networks.

A spatial information network (SIN) is composed of high Earth orbit (HEO) satellites, medium Earth orbit (MEO) satellites, low Earth orbit (LEO) satellites, near-space platforms, and ground terminals, forming a globally integrated information network primarily focused on communication, navigation, and remote sensing functions to enable diversified services worldwide. With advances in satellite payload technology and inter-satellite link capabilities, SINs have progressively evolved from single-function constellations—such as navigation constellations, communication mega-constellations, and remote sensing observation constellations—toward an integrated communication, navigation, and remote sensing paradigm [13]. Currently, the development of satellite constellations such as USPACE + AICO, China’s LEO-NA project, Starlink, OneWeb, and Telesat has positioned integration technology as the mainstream pathway for orbital constellation development. Accordingly, in conducting network topology analysis of SINs, this paper primarily considers LEO satellite constellations that perform integrated communication, remote sensing observation, and navigation enhancement functions. In addition, the satellite network layer includes MEO and HEO satellite constellations serving as information transmission supplements [14], alongside the ground user layer supporting tasks such as communication, navigation, and remote sensing, as illustrated in Figure 1.

(1) Satellite Network Layer

The satellite network layer primarily comprises a hybrid constellation network integrating low Earth orbit (LEO), medium Earth orbit (MEO), and high Earth orbit (HEO) satellites [14]. LEO satellites are mainly composed of constellations such as Starlink, OneWeb, Iridium, Globalstar, and China’s satellite network. Their orbital altitudes are predominantly concentrated between 500 and 1500 km, offering advantages such as short propagation delays, brief orbital periods, and low link losses. MEO satellites are primarily utilized for navigation and broadband relay services, with orbital altitudes mainly ranging from 2000 to 20,000 km, thereby balancing lower latency with broader coverage. HEO satellites, exemplified by geostationary Earth orbit (GEO) satellites, operate at an altitude of approximately 35,786 km. These satellites exhibit rotation synchronization with the Earth, maintain nearly constant viewing angles, and provide the largest coverage area.

**Figure 2:**
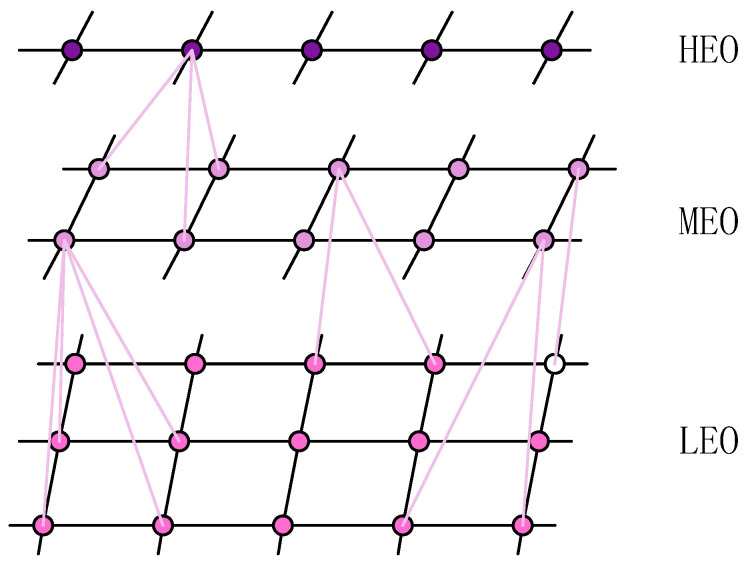
Satellite Network Topology.

Given the low cost and large-scale deployability of LEO satellites, this study adopts LEO satellites as the foundational platform for integrated communication, navigation, and remote sensing functions within the SIN architecture. MEO satellites are connected to LEO satellites, and HEO satellites are linked to MEO satellites, thereby supplementing the information transmission services provided by the LEO segment. The resulting network topology model is illustrated in Figure 2:

(2) Ground User Layer

Owing to the multifunctionality of SINs, the ground user layer exhibits diverse types and wide geographic distribution [15]. This study primarily considers three categories of users. First is communication users, encompassing individuals, devices, and ground stations that utilize satellite communication services. Their distribution is mainly concentrated in developed regions and densely populated areas. Second is remote sensing users, which consist of various observation nodes and ground stations responsible for receiving and processing remote sensing data. The observation nodes are assumed to be distributed proportionally between oceanic and terrestrial regions, with a focus on developed areas and key maritime shipping routes. Third is navigation users, comprising individuals and devices that rely on satellite navigation services. These users are distributed across both ocean and land according to a defined ratio, primarily guided by population density, while also accounting for navigation demands in remote and sparsely populated regions.

**Figure 3:**
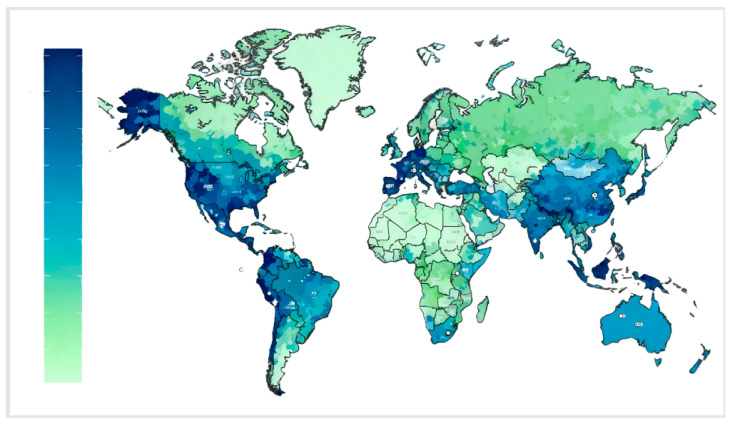
Population density distribution.

**Figure 4:**
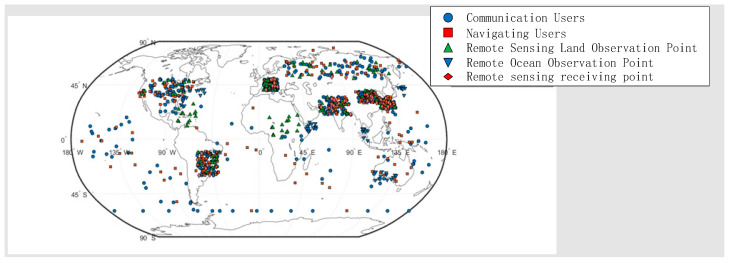
Ground Node Distribution.

As illustrated in Figure 3, the distribution of the ground user layer is inherently uneven. This imbalance directly leads to disparities in the demand placed on SINs across different regions. Therefore, when analyzing the network structure of SINs, it is essential to incorporate specific user distribution characteristics. Based on the distribution patterns of the three user types and integrating factors such as population density, a simulated distribution of the ground user layer is constructed, as depicted in Figure 4:

### 2.2. Problems and Challenges in Vulnerability Analysis of Spatial Information Networks

The main challenges currently confronting SINs are manifested in several key aspects:

First, the dynamic topology of satellite networks exhibits significant spatiotemporal characteristics. The high-speed orbital motion of core nodes—such as satellites—leads to continuous changes in inter-satellite and satellite–ground link connectivity, resulting in highly dynamic topological structures and inherent uncertainties. These dynamics give rise to strong spatiotemporal coupling properties. Traditional static topological analysis frameworks are inadequate for accurately capturing such variations, severely compromising the timeliness and accuracy of vulnerability assessments [16,17].

Second, the expanding scale of networks poses significant challenges to the computational complexity of topological analysis. The deployment of large low-orbit constellations and the construction of multi-layer space-based networks have led to a sharp increase in the number of satellite nodes, ground users, and payloads, forming large-scale topologies interwoven with vast numbers of nodes. Conventional graph-theoretic analysis methods suffer from exponential growth in computational data and low processing efficiency, rendering them inadequate for real-time vulnerability assessment and emergency decision-making requirements [18].

Third, the contradiction between topological orderliness and uneven user distribution emerges as a core issue in task-driven vulnerability analysis. During the design phase, SIN topologies generally adhere to well-ordered construction principles to ensure global communication efficiency and coverage integrity. However, in practical application scenarios, the distribution of ground user demand exhibits pronounced imbalances—user terminals in critical areas such as urban centers, battlefields, and disaster rescue sites are highly concentrated, leading to instantaneous surges in task requests, while user demand in remote regions remains sparse and dispersed. This mismatch between “designed orderliness” and “demand imbalance” directly results in an extremely uneven distribution of network task loads across topological nodes. Specifically, certain core nodes must bear task forwarding and data processing pressures far exceeding their design thresholds, thereby becoming potential high-vulnerability bottlenecks. Conversely, some edge nodes remain underutilized, operating in a lightly loaded state with low resource efficiency. Traditional task-driven vulnerability analysis methods largely rely on the assumption of uniform load distribution, making it difficult to accurately characterize the evolution of node vulnerability under such unbalanced load conditions. Consequently, these approaches fail to anticipate the cascading failure risks induced by node overload in advance and are unable to formulate differentiated hardening strategies accordingly. Ultimately, this limitation severely undermines the practical guidance value of vulnerability analysis [19].

In summary, to address the challenges outlined above—particularly the core issue of mismatch between topological orderliness and imbalanced user demand—this paper proposes a modeling framework based on multi-layer hypernetworks. The proposed approach integrates the ground user layer and the satellite network layer into a unified multi-layer space–ground integrated network. By employing hypernetwork theory, a task hypernetwork is constructed to characterize imbalanced task distributions from the perspective of the ground user layer. Three task-specific efficiency indicators are defined to support vulnerability assessment. Furthermore, based on the multi-layer hypernetwork structure, key node identification strategies—such as hypernetwork overlap degree and betweenness centrality—are developed to enable precise vulnerability analysis of SINs.

## 3. SIN Models Based on Multilayer Hypernetworks

In this section, the construction process of the multi-layer hypernetwork model for spatial information networks (SINs) is introduced. Subsequently, key concepts—including the network model definition, various types of task efficiency, and vulnerability assessment indicators—are systematically elaborated to clarify the analytical framework for vulnerability evaluation.

### 3.1. Building Multilayer Hypernet Model

According to Bretto’s definition of hypernetworks and their properties [20], in a conventional network topology, each edge can connect only two nodes. In contrast, within a hypernetwork, a hyperedge is capable of connecting an arbitrary number of nodes. The mathematical definition of a hypernetwork is as follows: a hypernetwork is defined as H=(V,E), where $V=\{v_{i}|i=1,2,\cdots ,n\}$ is a finite set of nodes and E is a finite set of edges, and edge $e_{j}$ is a subset of V, where $e_{j}\subseteq V$ and $e_{j}\neq \emptyset$. Furthermore, when hyperedges are associated with distinct weights, the hypernetwork can be extended to a weighted hypernetwork, denoted as H=(V,E,ω), where ω(e) represents the weights assigned to each hyperedge. These weights may correspond to various attributes such as strength, capacity, or task importance.

**Figure 5:**
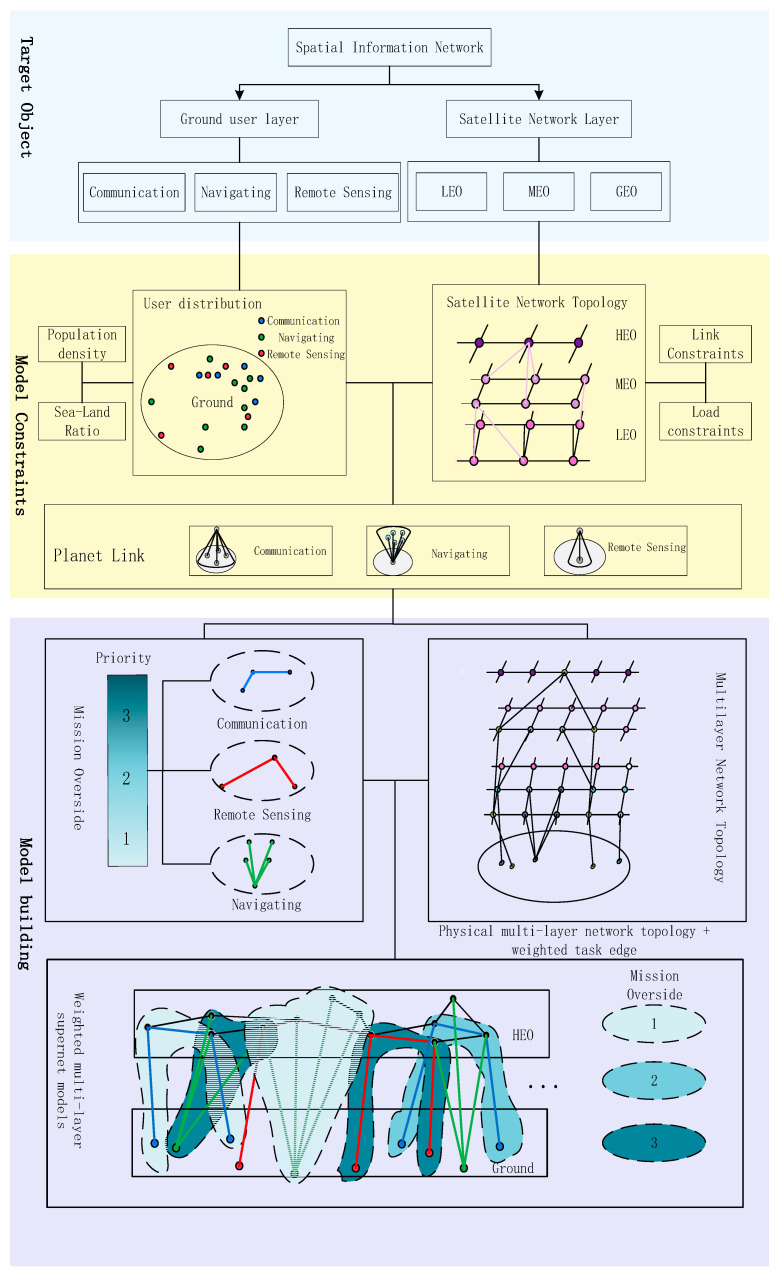
Multilayer Hypernet Model Building Process.

This section constructs a multi-layer hypernetwork model for SINs based on the SIN composition described in Section 2.1 and hypernetwork theory. First, the multi-layer physical network topology of the SIN is established, encompassing both the ground user layer and the satellite network layer. Next, task edge modeling is performed according to the task requirements of the SIN. Subsequently, task priorities are determined based on the geographic location information of task users. Finally, a task-weighted multi-layer hypernetwork model for SINs is developed. The overall construction process is illustrated in Figure 5.

#### 3.1.1. Physical Network Topology Model

As established in Section 2.1, the SIN is divided into two primary layers: the ground user layer and the satellite network layer. Using complex network graph theory, satellites and users are abstracted as nodes, while information transmission links are abstracted as edges connecting these nodes.

1. Ground User Layer

**Figure 6:**
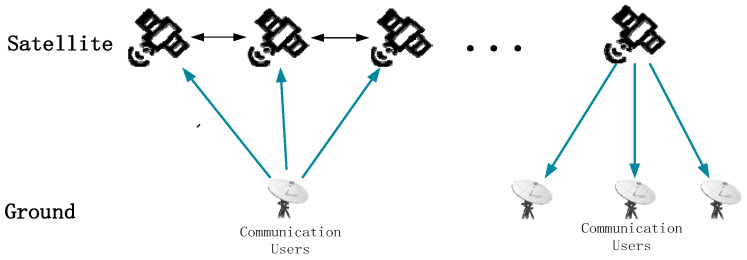
Communication Node Constraints.

**Figure 7:**
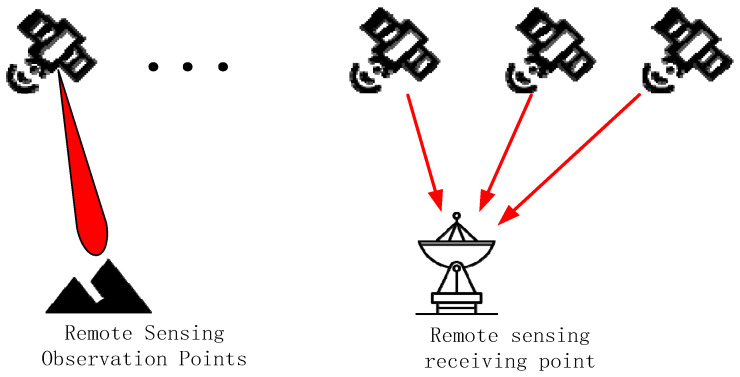
Remote Sensing Node Constraints.

**Figure 8:**
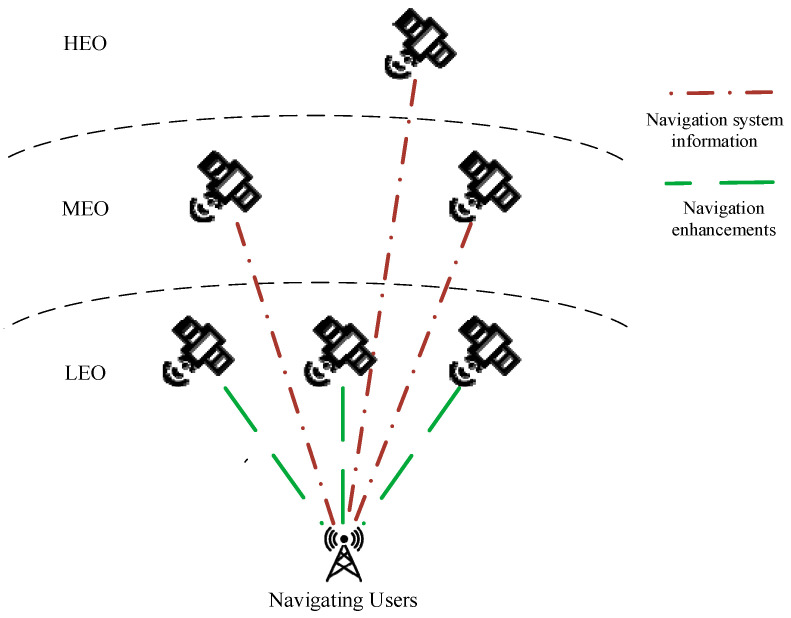
Navigation Node Constraints.

The ground user layer primarily consists of nodes categorized according to task types into communication nodes, remote sensing observation points, remote sensing receiving points, and navigation nodes. Given that this study focuses on low Earth orbit (LEO) satellites as the platform for implementing integrated communication and remote sensing functions, link constraints are established between ground user nodes and the LEO satellite layer within the satellite network layer. Accordingly, inter-layer links are constructed between the ground user layer and the satellite network layer, as illustrated in Figure 6, Figure 7 and Figure 8.

(1) Communication Nodes

Satellite communication can be abstracted as the process of information transmission between remote users via satellite nodes, as illustrated in Figure 6. Accordingly, link constraints are established between ground users and satellite nodes, forming the satellite–ground links that interconnect the ground user layer and the satellite network layer within the SIN.

(2) Remote Sensing Node

The entire remote sensing process can be abstracted as follows: first, a satellite node establishes an observation link with a ground observation point. Subsequently, the satellite node transmits the acquired remote sensing data through the satellite network path to a ground receiving point. This process is illustrated in Figure 7.

(3) Navigation nodes

Ground navigation users update their positioning information by receiving broadcast signals from navigation satellites. The navigation information originates from two sources: one is the dedicated navigation constellation, typically comprising medium Earth orbit (MEO) and high Earth orbit (HEO) satellite groups; the other is augmentation navigation information provided by low Earth orbit (LEO) satellites. This dual-source navigation information delivery process is illustrated in Figure 8.

2. Satellite Network Layer

The satellite network layer comprises a multi-layer satellite network topology integrating LEO, MEO, and HEO satellites, interconnected via laser inter-satellite links (ISLs). These links are primarily categorized into intra-layer links and cross-layer links.

First are the intra-layer links. Based on current mainstream research [21], this paper assumes that each satellite within the same layer maintains four inter-satellite links. Specifically, each satellite establishes permanent links with the two neighboring satellites on the same orbital plane (i.e., the preceding and following satellites). Additionally, a dynamic link is established with the nearest satellite in the adjacent, same-direction orbital plane. This dynamic link is periodically disconnected and re-established over time due to relative satellite motion.

Second are the cross-layer links. This study adopts a hierarchical inter-layer connection strategy, wherein the MEO layer establishes links to the LEO layer, and the HEO layer establishes links to the MEO layer. This approach effectively controls cross-layer transmission latency and reduces the communication load on LEO satellites. Considering the constraints of satellite resources and the dynamic nature of satellite network topologies, this paper defines that when a higher-layer satellite node establishes a link to a lower-layer satellite, it selects the four closest nodes within the lower layer to establish connections.

Accordingly, the SIN is defined as consisting of a finite set of nodes $V=\{v_{i}|i=1,2,\cdots ,n\}$, where each node represents an entity within either the ground user layer or the satellite network layer—such as satellites, ground observation points, or ground receiving stations. The edge set is denoted as $E^{G}=\{e_{j}^{G}|j=1,2,\cdots ,m\}$, where each edge $e_{j}^{G}$ represents a physical link between two entities, including satellite–ground links between the user layer and satellites, as well as inter-satellite links among satellites. The node set V together with the edge set E constitutes the physical network topology of the SIN. Thus, the physical network of the SIN is formally defined as:

(1) $$G=(V,E^{H})$$

#### 3.1.2. Task Hyper-Edge Model

**Figure 9:**
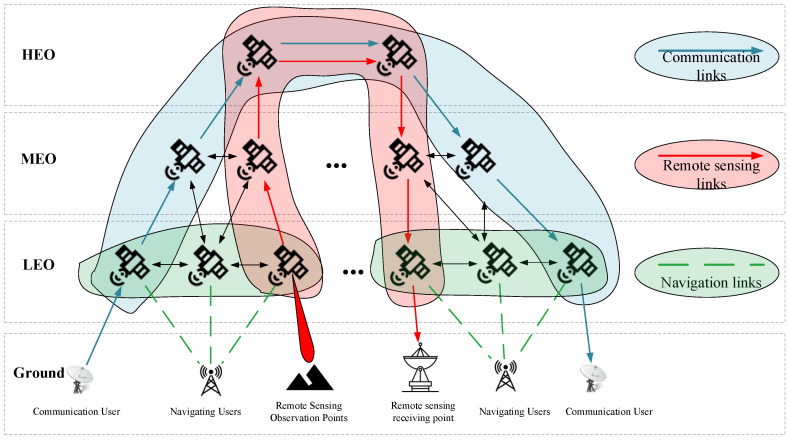
Task Ultra-Edge Model.

This paper primarily considers the performance of SINs in three types of tasks: communication, navigation, and remote sensing. Accordingly, each task type is formally defined as a hyper-edge model, as illustrated in Figure 9:

First, based on the hypernetwork theory introduced in Section 3.1, a task-based hyperedge set $E^{H}=\{e_{k}^{H}|k=1,2,\cdots ,l\}$ is constructed, where each hyperedge $e_{k}^{H}\in V$ and $e_{k}^{H}\notin \emptyset$ is defined as the set of nodes involved in executing a specific task. Accordingly, multiple task hyperedges of different types collectively form the task hypernetwork, which is formally defined as follows:

$$H=(V,E^{G})$$

Here, $e_{k}^{H}$ denotes the k-th task hyperedge in the task hypernetwork H. Depending on the type of task, each hyperedge is specifically defined as follows:

1. Communication Task

First, by considering both the information processing latency at nodes and the transmission latency of inter-satellite links, a constrained A* heuristic search algorithm [16] is employed to plan the shortest-latency path from the source node to the target node. The source and target nodes here refer to communication nodes within the ground user layer. The information transmission task hyperedge is constructed by aggregating all nodes along the planned path from the source node to the target node, which together form the hyperedges ${e_{com}}_{j}^{H}$.

(2) $${e_{com}}_{j}^{H}=\{v_{source},v_{i}\cdots ,v_{t\arg et}\},j\in \{1,2,\cdots ,n_{com}\},v_{i}\in G$$

where ${e_{com}}_{j}^{H}$ represents the jth hyper edge, that is, the jth information transmission task, $n_{com}$ is the total number of communication tasks, $v_{source}$ is the source node, $v_{t\arg et}$ is the destination node, i belongs to the serial number that contains the node in the path, and G is the physical network topology.

2. Remote Sensing Task

First, by accounting for both node information processing latency and inter-satellite link transmission latency, a constrained A* heuristic search algorithm [22] is employed to plan the shortest-latency path from the source node to the target node. In this context, the source node refers to a remote sensing observation point, and the target node refers to a remote sensing receiving node, both belonging to the ground user layer. The remote sensing information transmission task hyperedge is constructed by aggregating all nodes along the planned path from the source node to the target node, which together form the hyperedge ${e_{\mathrm{rs}}}_{j}^{H}$.

(3) $${e_{rs}}_{j}^{H}=\{v_{obs},v_{i}\cdots ,v_{rec}\},j\in \{1,2,\cdots ,n_{rs}\},v_{i}\in G$$

Here, ${e_{rs}}_{j}^{H}$ denotes the j-th remote sensing hyperedge, representing the j-th remote sensing task. Let $n_{rs}$ be the total number of remote sensing tasks. In this context, $v_{obs}$ is the observation node, $v_{rec}$ is the receiving node, $v_{i}$ is indexes the sequence of nodes along the path, and G represents the physical network topology.

3. Navigation task

In this study, the navigation augmentation effect is primarily considered for low Earth orbit (LEO) satellites. It can be abstracted as the set of LEO satellites whose signals can be received at the location of a ground navigation user node, thereby constituting LEO satellite-based navigation augmentation. Accordingly, the navigation task hyperedge is constructed as the set of all LEO satellites within the visibility range of the ground user node, denoted a ${e_{nav}}_{j}^{G}$.

(4) $${e_{nav}}_{j}^{H}=\{v_{i_{1}},\cdots ,v_{i_{m}}\},j\in \{1,2,\cdots ,n\},v_{i}\in H$$

where m is the number of low Earth orbit satellites within the visibility range of the navigation user, and n is the total number of navigation tasks.

#### 3.1.3. Priority

When analyzing the vulnerability of SINs, the performance outcomes may vary depending on the specific areas of focus. Therefore, to enable a more accurate analysis of SINs in practical application scenarios, this study categorizes task regions into hotspot areas, general areas, and non-focused areas. Based on this regional classification, corresponding weights are assigned to the tasks, thereby yielding more targeted vulnerability analysis results.

(5) $$\mathrm{Pr}=\left\{\begin{array}{l} 3,(lat_{i},lon_{i})\in region_{hot}\\ 2,(lat_{i},lon_{i})\in region_{normal}\\ 1,(lat_{i},lon_{i})\in region_{not-f} \end{array}\right.$$

(6) $$\omega_{i}=\left\{\begin{array}{l} 1,\mathrm{Pr}=3\\ 0.8,\mathrm{Pr}=2\\ 0.5,\mathrm{Pr}=1 \end{array}\right.,i=(1,2,\cdots ,m)$$

Here, $region_{hot},region_{normal},region_{not-f}$ represent hotspot areas, general areas, and non-focused areas, respectively. Typically, these area sets are selected based on predefined latitude and longitude ranges. Let $\left(lat_{i},lon_{i}\right)$ denote the latitude and longitude of the source node for an information transmission task or the location of a navigation user node, and let m be the total number of tasks.

### 3.2. Basic Definition

#### 3.2.1. Task Efficiency

When discussing vulnerability analysis of SINs, it is essential to select appropriate quantitative metrics to evaluate the performance of various tasks. Traditional network efficiency [12] is typically computed directly from the network topology by determining the shortest paths between all node pairs. This approach can comprehensively measure the efficiency of information transmission across the entire network. However, in practical scenarios, SINs face uneven task distributions and diverse task types. Consequently, traditional network efficiency is inadequate for conducting targeted vulnerability analysis of SINs. To address this limitation, this paper proposes three task efficiency (TE) formulas tailored to different task types, along with a quantitative vulnerability metric.

(1) Communication efficiency

In communication tasks, the primary focus is on the latency of the communication information path. In the network topology, path latency is abstracted as the transmission latency of edges and the forwarding latency of nodes. We define the transmission latency of an edge as $2t_{0}$ and the forwarding latency of a node as $t_{0}$.

Based on the derivation of the shortest physical path for satellite–ground information transmission in SINs, the basic transmission path is defined as: source ground node → one LEO satellite → destination ground node. This path involves no additional relay satellites and represents the minimum-link path for information delivery in SINs. Its total delay consists of five identical basic delay units: source node forwarding delay $t_{0}$, satellite-to-ground downlink transmission delay $t_{0}$, ground-to-satellite uplink transmission delay $t_{0}$, ground-to-satellite uplink 2 transmission delay $t_{0}$ and destination node reception delay $t_{0}$. Thus, the total delay is given by $5t_{0}$. Therefore, the optimal transmission latency for information transmission tasks should be $5t_{0}$. We define the communication information transmission efficiency as follows:

(7) $$TE(e_{com}^{H})=\omega_{i}\times e^{\sigma_{com}(5t_{0}-t)}$$

Among them, t denotes the shortest delay from the source node to the target node within the task hyperedge, and $\sigma_{com}$ is the communication transmission coefficient.

(2) Remote Sensing Efficiency

In remote sensing information transmission tasks, the primary focus is also on the latency of the information transmission path. Within the network topology, path latency is abstracted as the transmission latency of edges and the forwarding latency of nodes. We define the transmission latency of an edge as $2t_{0}$ and the forwarding latency of a node as $t_{0}$. A complete remote sensing information transmission task typically involves a path from the source node (observation point) through at least one satellite node to the destination node (receiving station). Accordingly, the optimal transmission latency for such tasks should be the minimum achievable path delay, denoted as $5t_{0}$. We define the remote sensing information transmission efficiency TE as follows:

(8) $$TE(e_{re}^{H})=\omega_{i}\times e^{\sigma_{rs}(5t_{0}-t)}$$

Among them, t denotes the shortest delay from the source node to the target node within the task hyperedge, and $\sigma_{rs}$ is the remote sensing transmission coefficient. The remote sensing transmission coefficient is a normalized correction factor that quantifies information transmission loss, bit error rate (BER), and processing efficiency in remote sensing tasks, with a value range of (0, 1]. In this study, based on the actual transmission characteristics of remote sensing satellites in SINs, this coefficient is calibrated as 0.95 through simulation experiments. It is employed to convert the delay ratio into a remote sensing task efficiency value with a unified dimension, thereby enabling comparative analysis with communication and navigation efficiency indicators.

(3) Navigation efficiency

Navigation efficiency is applied to characterize the navigation augmentation capability of low Earth orbit (LEO) satellites. The primary focus is on the number of LEO satellites within the visible range of ground navigation users: a larger number of visible satellites provides more navigation augmentation information. Furthermore, to ensure an effective navigation augmentation effect, this paper defines navigation efficiency as optimal when the number of visible satellites reaches seven. This definition is grounded in the Geometric Dilution of Precision (GDOP) theory of satellite navigation: when the number of LEO satellites within the visibility range of a ground navigation node is equal to or greater than seven, high-precision three-dimensional position and time solutions can be achieved, the GDOP value tends to minimize and stabilize, and the accuracy and reliability of navigation positioning reach an optimal level. Accordingly, navigation efficiency is defined as follows:

(9) $$TE(e_{nav}^{H})=\left\{\begin{array}{l} 0,m=0\\ \omega_{i}\times e^{\alpha (m-7)},0<m\leq 7\\ 1,m>7 \end{array}\right.$$

where α is the navigation coefficient, and m is the number of visible satellites at the navigation node. Accordingly, the overall task efficiency of the entire SIN is defined as follows:

(10) $$TE(SIN)=\frac{\sum_{e_{i}^{H}\in E^{H}}\omega_{i}\times TE(e_{i}^{H})}{\sum_{j=1}^{\left|E^{H}\right|}\omega_{j}}$$

Here, A denotes the number of hyperedges, and the TE of the SIN represents the average value of the sum of task efficiencies across all hyperedges. From the efficiency definitions for communication and remote sensing tasks, task efficiency is determined by the difference between the actual transmission delay and the ideal minimum delay. Specifically, the closer the actual transmission delay is to the ideal value, the closer the TE value approaches 1; if the path is disrupted, TE equals 0. For navigation tasks, efficiency is directly proportional to the number of visible satellites. When the number of visible satellites reaches its maximum, the TE value remains at 1.

The three types of mission efficiency indicators defined above provide a foundation and quantifiable benchmark for evaluating communication, navigation, and remote sensing services. It is important to note that this represents a standardized simplification intended for comparative vulnerability analysis. In real-world systems, task requirements may be more complex—involving factors such as multiple quality of service (QoS) levels or interdependencies among tasks. The indicators proposed in this study aim to establish a unified basis for vulnerability quantification. Their simplicity and clarity facilitate the identification of the influence mechanism of “topology–task” coupling on network vulnerability under controlled variable conditions. The introduction of more refined task models remains a key direction for future extensions of this framework.

#### 3.2.2. Vulnerability Indicators

SINs are designed to accomplish various types of tasks in order to meet user demands. When an SIN is disrupted or subjected to attacks, its task efficiency is severely affected. For network operators and maintainers, a critical question arises: which components are the weak points in the topology, and which nodes, if attacked, could trigger large-scale collapse? Abstractly, this translates into a mathematical characterization of the extent to which TE(SIN) degrades under adversarial conditions. Accordingly, vulnerability is defined as:

(11) $$Vab=\frac{\left(TE_{t_{0}}(SIN)-TE_{t}(SIN)\right)}{\left(t-t_{0}\right)}$$

Here, $t_{0}$ is the initial state, t is when it is subjected to a certain degree of damage, where the time is measured in units of time measured by the number of attacks. Therefore, a greater degree of decline indicates higher vulnerability, meaning that the SIN is less capable of satisfying user demands when subjected to that particular type of attack.

In this paper, the vulnerability analysis of SINs focuses on leveraging the characteristics of multi-layer hypernetworks to identify critical satellite nodes associated with user distribution. Subsequently, node removal strategies are employed to examine the changes in task efficiency when the SIN faces targeted attacks, thereby uncovering key nodes. Hardening measures are then implemented for these identified nodes, and the vulnerability levels before and after hardening are compared to demonstrate the effectiveness of the proposed approach in reducing the vulnerability of SINs.

#### 3.2.3. Task Completion Rate

This metric is designed to analyze task efficiency in SINs with varying satellite scales under different task sizes within the multi-layer hypernetwork model. To assess task efficiency consistently across different task sizes and satellite scales, the task completion rate is defined as the ratio of actual task efficiency to the total task efficiency. The formula is as follows:

(12) $$TEC(SIN)=\frac{TE{\left(SIN\right)}_{ture}}{TE{\left(SIN\right)}_{\max}}$$

Among them, $TE{\left(SIN\right)}_{ture}$ represents the actual task efficiency under the current task size and satellite scale, while $TE{\left(SIN\right)}_{\max}$ denotes the total task efficiency when all tasks under the current task size are completed. This formulation enables a more intuitive representation of the relationship between task size and satellite scale in SINs.

### 3.3. Task Efficiency and Vulnerability Analysis Method

**Figure 10:**
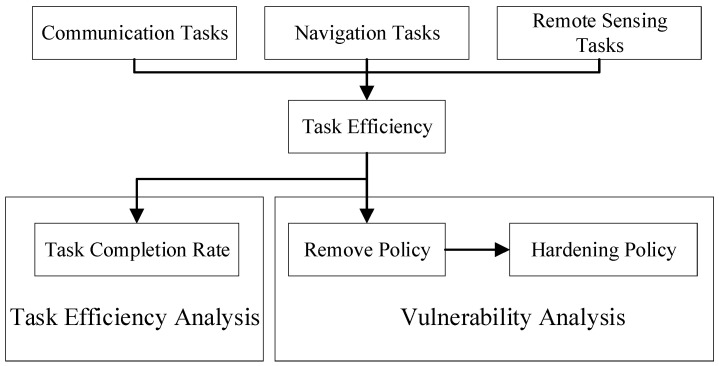
Task Efficiency and Vulnerability Analysis Method.

To enable vulnerability analysis of SINs in multi-task scenarios, the primary focus of task efficiency is on how the two key dimensions—satellite scale and task scale—affect task execution performance. Therefore, a unified metric, the task completion rate, is needed to accurately characterize these trends. Vulnerability analysis for SINs can be divided into two scenarios: (a) node removal strategy, which simulates satellite node failures caused by deliberate attacks or random failures; and (b) preemptive hardening strategy, which simulates the protection of critical nodes before an attack occurs, as a defensive measure. These two scenarios are illustrated in Figure 10.

#### 3.3.1. Removal Strategies

This paper discusses six node removal strategies based on network structural characteristics. The first three are commonly used standard node removal strategies: random node removal (RE), single-layer degree-centrality-based removal (SD), and single-layer betweenness-centrality-based removal (SB). The next two are multi-layer hypernetwork-based strategies: degree-centrality-based removal (MD) and betweenness-centrality-based removal (MB). Finally, the hypernetwork overlap removal strategy (HO) is proposed based on hypernetwork characteristics. The focus of this study is on the vulnerability of the satellite network layer within the SIN; therefore, the nodes selected for removal are satellite nodes.

**RE Removal Strategy**: A satellite node is randomly selected from the network for removal in each attack step, serving as a baseline for comparison.

**SD Removal Strategy** [23]: Traditional degree centrality is computed based solely on the satellite network layer topology, identifying nodes with the highest degree as critical. This strategy prioritizes the removal of high-degree nodes, aiming to disrupt the local connectivity of the network.

**SB Removal Strategy** [24]: Betweenness centrality measures the extent to which a node acts as a “bridge” along the shortest paths between other nodes in the network. Nodes with high betweenness centrality exert strong control over global information flow. This strategy targets high-betweenness nodes to disrupt long-distance paths across the network.

**MD Removal Strategy**: Under the multi-layer network framework, the topology incorporates satellite–ground links. Unlike traditional degree centrality computed solely from the satellite layer, the inclusion of unevenly distributed ground user nodes introduces an imbalance factor into the degree centrality of satellite nodes. This enables the metric to reflect actual task requirements when measuring local connectivity. The MD strategy prioritizes the removal of nodes with high multi-layer degree centrality to identify key nodes affecting local connectivity.

**MB Removal Strategy**: Similarly, under multi-layer network conditions, the inclusion of satellite–ground links differentiates this betweenness centrality calculation from traditional approaches. The uneven distribution of ground user nodes introduces an imbalance factor into the betweenness centrality of satellite nodes, allowing the metric to better capture vulnerability variations arising from differences in distribution characteristics. The MB strategy prioritizes the removal of nodes with high multi-layer betweenness centrality.

**HO Removal Strategy**: In the weighted multi-layer hypernetwork model, where each task corresponds to a hyperedge, the hypernetwork overlap of a node measures how frequently it appears across multiple high-priority task hyperedges. This strategy prioritizes the removal of nodes that “cover the most high-priority tasks”, thereby maximizing the scope of task failure. The hypernetwork overlap importance score So(v) for node v is defined as:

(13) $$So(v)=\sum_{\tau \in E_{v}^{H}}\left(TE(\tau )\times \frac{\mathrm{Pr}(\tau )}{\max_{\tau \prime \in E^{H}}\mathrm{Pr}(\tau \prime )}\right)$$

where $E_{v}^{H}$ is the set of task hyperbounds involved in section v, TE(τ) is the task efficiency of task τ, and Pr(τ) is the priority of task τ. Therefore, each execution of the removal policy is a destruction target of $v^{*}={\mathrm{arg max}}_{v\in G_{sat}}(So(v))$, where $G_{sat}$ represents the physical network topology of the satellite network layer.

The complete procedure for calculating So(v) is formalized in Algorithm 1. The core idea is to identify and rank nodes based on their aggregate contribution to high-value, high-efficiency tasks.
**Algorithm 1.** Calculate Hypernetwork Overlap (So(v))Input: Weighted multi-layer hypernetwork model H = (V, E^H, ω), Satellite node physical topology G_sat ⊆ VOutput: Dictionary Scores containing Hypernetwork Overlap score for each satellite node1: procedure Calculate Hypernetwork Overlap (H, G_sat)2: Scores ← empty dictionary             # Initialize score dictionary3: E^H ← set of all task hyperedges in H4: T_max ← max_{τ ∈ E^H} Pr(τ)           # Find global max task priority5: for each node v in G_sat do6:    score_v ← 0                  # Initialize score for node v7:    E_v^H ← {τ ∈ E^H | v ∈ τ}            # Find all hyperedges containing v8:     for each hyperedge τ in E_v^H do9:      w ← Pr(τ)/T_max                 # Calculate normalized priority weight10:         score_v ← score_v + TE(τ) × w        # Accumulate weighted task efficiency11:        end for12:     Scores [v] ← score_v # Store final score for node v13: end for

#### 3.3.2. Hardening Strategy

Hardening, as a preventive defense measure, can effectively reduce the vulnerability of SINs and must be considered during the initial network design and implementation phase to mitigate potential future attacks. However, SINs are required to handle a large number of heterogeneous task demands, and the significant imbalance in task distribution imposes heavy loads on certain nodes and edges within the network topology. Once such high-load nodes and edges are damaged, the overall task execution efficiency of the network is severely affected. Therefore, developing an effective hardening strategy and reinforcing critical nodes in advance can proactively defend against potential risks and substantially reduce the vulnerability of SINs.

The node removal strategies discussed in Section 3.3.1 determine the order in which nodes are destroyed under different attack scenarios. By reversing this order, a corresponding node protection sequence can be derived, leading to the formulation of five hardening strategies: SD Hardening Strategy, SB Hardening Strategy, MD Hardening Strategy, MB Hardening Strategy, and HO Hardening Strategy. The rationale for selecting these five strategies is based on their alignment with the removal strategies, the comprehensiveness of topological analysis dimensions, and experimental feasibility:

① Six node removal strategies are proposed in this study. Among them, the random removal (RE) strategy represents untargeted, non-predictable disruptions, and hardening randomly selected nodes lacks practical engineering significance. Therefore, the remaining five targeted removal strategies are correspondingly transformed into five targeted hardening strategies.

② The five hardening strategies comprehensively cover three core dimensions of SIN topological analysis: single-layer network topological features (SD, SB), multi-layer network topological features (MD, MB), and hypernetwork task correlation features (HO). This enables a systematic comparison of hardening effectiveness across different analytical dimensions and highlights the superiority of the HO hardening strategy proposed in this study.

③ The five hardening strategies are both representative and innovative within the field of SIN vulnerability protection, without introducing redundancy. The selected number of strategies balances experimental comprehensiveness and result comparability, avoiding the difficulty of distilling clear conclusions that may arise from an excessive number of strategies.

This paper applies the aforementioned hardening strategies to reinforce a certain number of nodes. To quantitatively evaluate the hardening effect, the area difference (AD) of the task efficiency curves before and after network hardening under the same removal strategy is adopted. The AD is defined as follows:

(14) $$AD=\int_{t_{0}}^{t}TE_{t}{\left(SIN\right)}_{harden}dt-TE_{t}{\left(SIN\right)}_{original}dt$$

Among them, $TE_{t}{\left(SIN\right)}_{harden}$ represents the task efficiency curve as a function of the removal step after hardening, while $TE_{t}{\left(SIN\right)}_{original}$ denotes the task efficiency curve under the same removal strategy without hardening. A larger AD value indicates a slower decline in task efficiency after hardening, signifying a better hardening effect.

## 4. Experimental Comparison Analysis

In this section, the case study parameter settings are first introduced. Subsequently, a comparative analysis of the effects of the six removal strategies is conducted, examining the characteristics of each strategy and validating the advantages of the removal strategy proposed in this paper. Finally, five hardening strategies are applied to reinforce the network topology, and the reinforced networks are subjected to the proposed removal strategy to evaluate and compare the hardening effectiveness of the different strategies.

### 4.1. Scene and Parameter Settings

To ensure the rationality, comprehensiveness, and reproducibility of the experiments, this section defines the core parameters of the physical network (including both the satellite network layer and the ground user layer) as well as the mission network. Based on the characteristics of actual satellite constellation deployments and user demand distributions, multiple sets of variable-controlling experiments are designed to investigate the influence of three key factors—satellite scale, mission scale, and orbit configuration—on the experimental results. The specific parameter settings are as follows.

In this section, the basic parameters are defined for both the physical network and the mission network. The physical network is a multi-layer network topology constructed from the ground user network and the satellite network at a given time instant. Based on population density [25] and land–sea distribution, multiple types of ground nodes—including communication nodes, observation nodes, receiving nodes, and navigation nodes—are established. Table 1 presents the core parameters of the satellite constellation, specifying the orbital altitude, orbital inclination, number of orbital planes, number of satellites per orbital plane, and phase factor for LEO, MEO, and HEO satellites [26,27]. These parameter settings are informed by the deployment characteristics of current mainstream satellite constellations, such as Starlink and OneWeb. Specifically, LEO satellites are placed at an orbital altitude of 1200 km (balancing low link loss and coverage), with 10 orbital planes and 10 satellites per plane. MEO satellites operate at an altitude of 20,000 km, primarily responsible for relay transmission functions. HEO satellites are positioned in geosynchronous orbit (35,786 km) to achieve wide-area coverage. The distinct parameters of the three satellite types reflect their functional roles within the SIN, thereby providing a foundation for subsequent analysis of the mission contributions of satellites in different orbits.

Table 2 defines the mission parameters corresponding to the three core SIN tasks—communication, navigation, and remote sensing—and specifies the types and quantities of ground nodes involved in each task. Specifically, there are 50 communication nodes and 50 navigation nodes, along with 10 remote sensing observation nodes and 5 receiving nodes. Considering the concentrated nature of remote sensing tasks and the specialized requirements of data processing, the node quantities are set to balance task diversity with experimental feasibility. These configurations provide standardized task scenarios for subsequent task efficiency quantification and vulnerability analysis.

Table 3 presents the regional priority parameter settings. In consideration of the inherent imbalance in actual user demand, the task area is divided into three categories: hotspot areas (priority 3), general areas (priority 2), and non-focused areas (priority 1), with the latitude and longitude ranges clearly defined for each category. Hotspot areas correspond to regions with dense populations and concentrated task demands, general areas represent regions with moderate demand levels, and non-focused areas comprise the remaining regions. This classification enables the quantification of task priority differences, thereby making the key node identification in the subsequent HO strategy more aligned with practical application scenarios [28].

Table 4 provides detailed information on ground node priorities (excerpt), including the latitude, longitude, priority level, and node type for each ground node. This table serves as the core data foundation for task priority assignment and task hyperedge construction, ensuring the accuracy of subsequent task efficiency calculations and key node identification [29].

**Figure 11:**
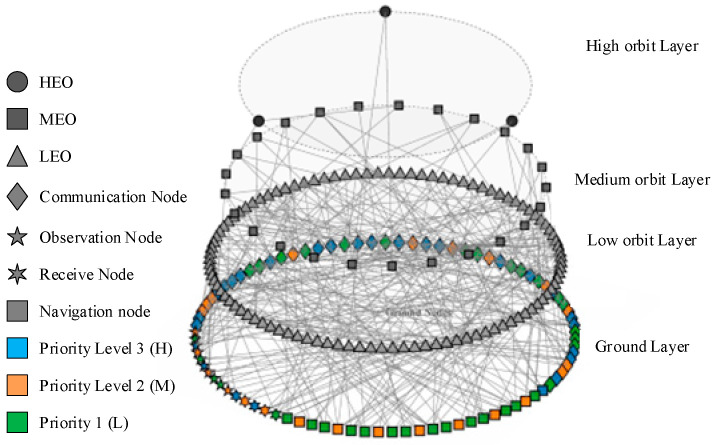
Network Topology Diagram.

Based on the aforementioned parameter settings, the network topology between satellite and ground nodes is obtained through simulation, as illustrated in Figure 11.

To comprehensively analyze the relationship between satellite network scale and mission scale, this study defines the experimental validation data parameters as shown in the table. In the table C, R, N, and T denote the number of communication missions, remote sensing missions, navigation missions, and total missions, respectively. LEO, MEO, HEO represent the number of low Earth orbit, medium Earth orbit, and high Earth orbit satellites, while O-L, O-M, and O-H indicate the number of orbital planes for LEO, MEO, and HEO satellites, respectively. Through experimental groups 1-1 to 5-5, the task efficiency performance and vulnerability of different satellite scales under varying mission scales are systematically characterized, as presented in Table 5.

In order to ensure the reproducibility of the simulations, the values of the coefficients in the basic definitions are specified in Table 6 below:

### 4.2. Task Efficiency Analysis

**Figure 12:**
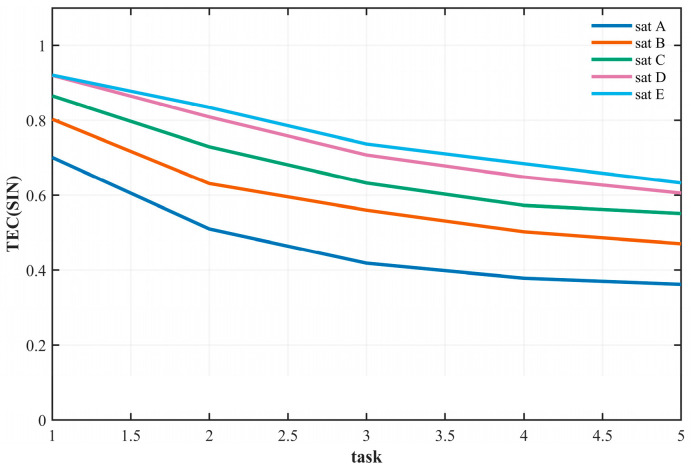
Task Completion Efficiency Analysis.

Task efficiency is a core metric for evaluating the performance of SINs. This section focuses on analyzing the impact of satellite scale, mission scale, and orbit type on task efficiency. Based on Figure 11 and Figure 12, the rationality of the proposed task efficiency quantification method is verified.

Figure 12 presents the variation in task completion efficiency under different satellite scales and mission scales. The abscissa represents the mission scale, where mission scales 1 through 5 correspond to 300, 700, 1100, 1500, and 1900 tasks, respectively. The ordinate denotes the task completion efficiency (TE). Curves A through E correspond to satellite scale groups 1-1 to 1-5 as defined in Table 5.

The key observations are as follows: First, mission scale has a negative impact on task efficiency. Regardless of satellite scale, efficiency decreases as the mission scale increases. Second, satellite scale has a positive effect on task efficiency: under the same mission scale, larger satellite scales yield higher task efficiency. Moreover, the efficiency of small-scale satellites is more sensitive to changes in mission scale. Additionally, as satellite scale expands, the efficiency gain exhibits a diminishing marginal effect—once node redundancy reaches a certain level, the contribution of additional satellites to load sharing becomes limited.

**Figure 13:**
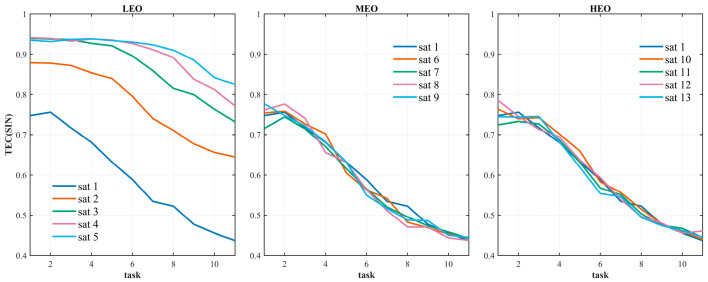
Track Type Analysis.

To further analyze the relationship between mission efficiency and the satellite scales of LEO, MEO, and HEO, this section defines the mission scales and satellite scales as presented in Table 7:

Figure 13 presents a comparative analysis of the impact of different orbital satellite scales on mission efficiency, with separate subplots for LEO, MEO, and HEO satellites. The abscissa represents the mission scale, and the ordinate denotes mission completion efficiency. Each curve in the subplots corresponds to variations in the number of satellites within a single orbit type, while the satellite counts in the other orbits remain fixed, as specified in Table 6.

The key observations are as follows: First, LEO satellites have the most significant impact on mission efficiency. Increasing the number of LEO satellites substantially improves efficiency—particularly at smaller mission scales—whereas changes in the number of MEO and HEO satellites have only a marginal effect. This finding aligns with the functional roles of the three orbit types: LEO satellites serve as the core execution nodes, while MEO and HEO satellites primarily provide supplementary communication functions. Second, the contribution of MEO satellites to efficiency is slightly higher than that of HEO satellites, owing to their orbital advantage in assisting LEO relay transmissions. In contrast, HEO satellites, due to their higher altitude and correspondingly larger latency, have a limited impact on mission efficiency. These results further reinforce the conclusion that LEO satellite constellations constitute the key protection cluster within SINs.

### 4.3. Comparison Analysis of Removal Policies

By comparing the experimental results of six node removal strategies—RE, SD, SB, MD, MB, and HO—this section analyzes the declining trend of task efficiency under different strategies and evaluates their impact on task performance. The superiority of the proposed HO removal strategy in identifying critical nodes is verified. Furthermore, the influence of satellite scale and mission scale on the effectiveness of the removal strategies is clarified.

(1) Satellite Size

**Figure 14:**
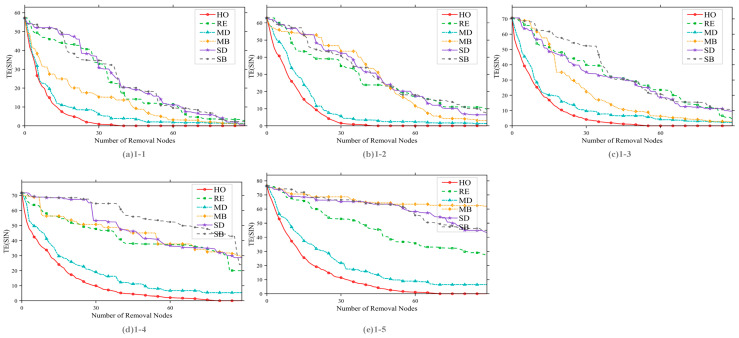
Remove Policy Comparison Analysis.

Figure 14 presents the mission efficiency curves for the six removal strategies under different satellite scales. The abscissa represents the number of removed nodes (fixed at 90), and the ordinate denotes mission completion efficiency. The curves correspond to the six strategies—RE, SD, SB, MD, MB, and HO—across experimental groups A to E, which represent different satellite scales as defined in Table 5.

The key observations are as follows: First, the HO removal strategy consistently exhibits the best performance, achieving the greatest reduction in mission efficiency regardless of satellite scale. This indicates that HO can accurately identify core nodes that critically affect network performance—nodes that participate simultaneously in multiple types of high-value tasks, leading to widespread task failures upon their removal. Second, as satellite scale increases, the efficiency gap between the HO strategy and other strategies becomes more pronounced. This suggests that under larger and more complex network task coupling, the limitations of single-layer and multi-layer strategies that do not incorporate task associations become increasingly evident.

(2) Task Scale

**Figure 15:**
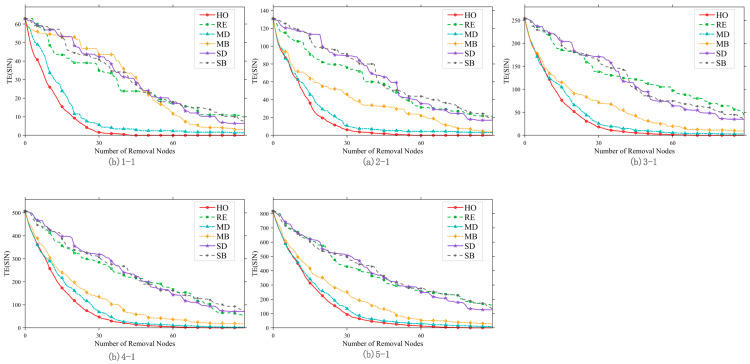
Remove Policy Comparison Analysis (Task Scale).

Figure 15 presents the comparison curves of the six removal strategies under different task scales. The abscissa represents the number of removed nodes, and the ordinate denotes task completion efficiency. The experimental groups correspond to increasing task scales as defined in Table 5.

The results indicate that the HO removal strategy consistently achieves optimal and stable performance across all task scales. However, as the task scale increases, its discriminative ability weakens slightly, and the efficiency gap between HO and the MD and MB strategies tends to narrow. This convergence is attributed to the saturation of satellite node task participation, which leads to the convergence of node characteristics under high task loads. Nevertheless, the HO strategy still outperforms all other strategies, further verifying its effectiveness in identifying critical nodes under varying task conditions.

### 4.4. Hardening Policy Comparison Analysis

**Figure 16:**
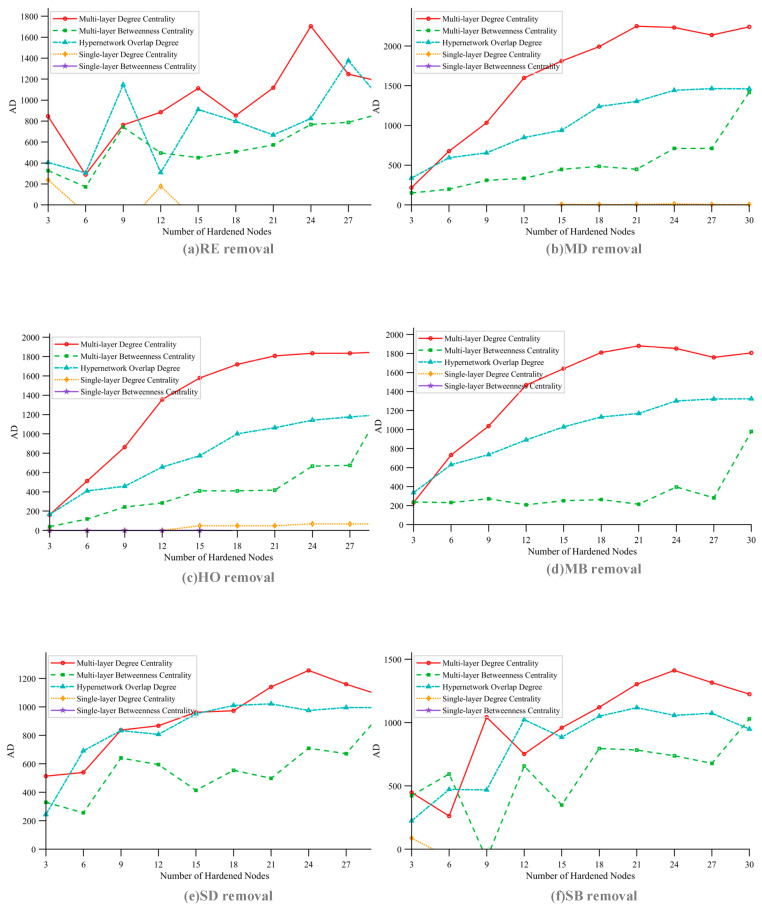
Hardening Policy Comparison.

**Figure 17:**
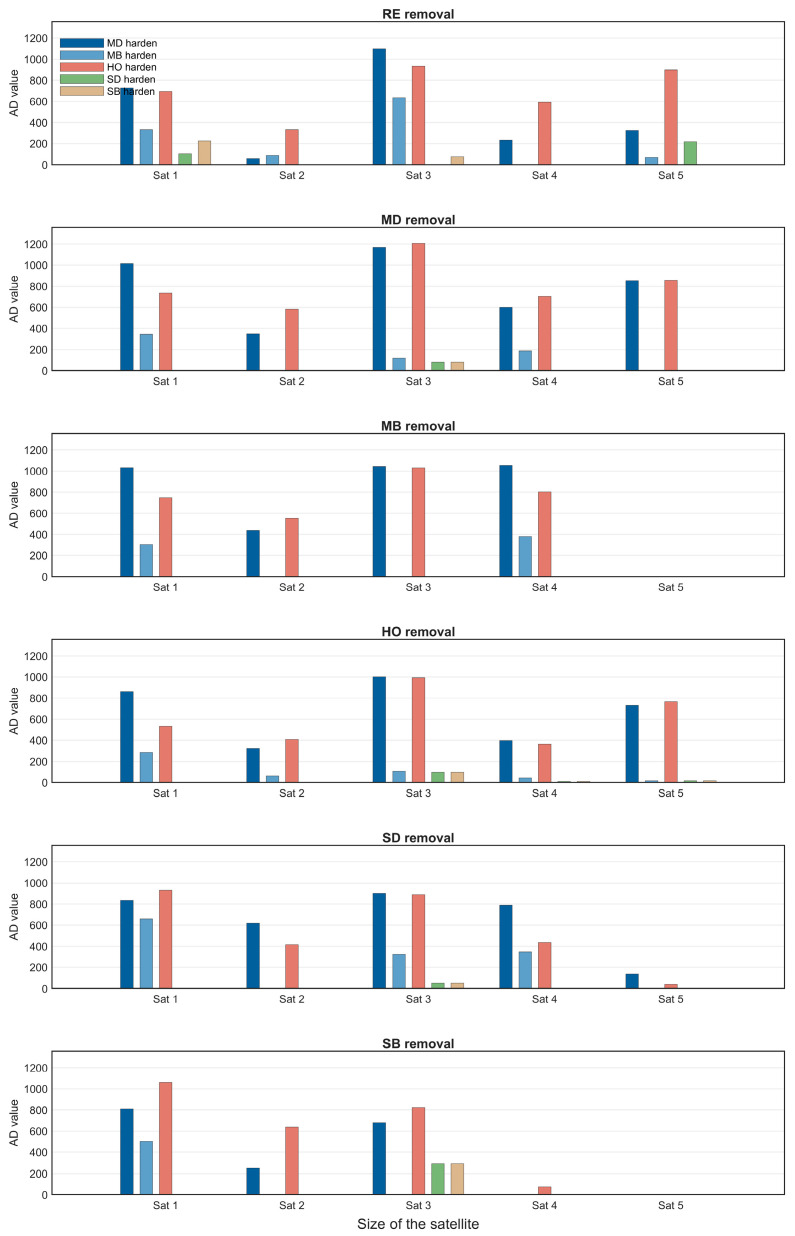
Comparison of hardening effects of different satellite sizes.

Hardening strategies are core defensive measures for reducing the vulnerability of SINs. This section compares the defense effectiveness of five hardening strategies—SD, SB, MD, MB, and HO—using Figure 16 and Figure 17. The influence of satellite scale on hardening strategy performance is analyzed, the optimal strategy selection under different scenarios is clarified, and the superiority of the HO hardening strategy is verified.

Figure 16 presents a comparison of the defensive effectiveness of the five hardening strategies for satellite scale 1 (corresponding to group 1-1 in Table 5). Hardening effectiveness is quantified by the area difference (AD) of the efficiency curves, where a larger AD value indicates better performance. The results show that multi-layer network hardening strategies (MD, MB, HO) significantly outperform single-layer strategies (SD, SB), as the former integrate multi-layer topology and task correlation to protect core nodes more effectively. The hardening effect varies under different removal strategies: when facing RE, SD, and SB removal, HO and MD exhibit similar effectiveness; under MD and MB removal, MD outperforms HO. However, overall, the HO strategy demonstrates the most stable performance across different attack scenarios.

To systematically evaluate the effectiveness of different hardening strategies across multiple scenarios, this section fixes the number of hardened nodes at 20 and compares the performance of each hardening strategy under four typical removal strategies—RE, MD, MB, and HO—under the same task scale, across satellite scales 1 to 5.

Figure 17 presents a comparison of the three optimal hardening strategies—MD, MB, and HO—under different satellite scales, with each subplot corresponding to a different removal strategy. The key conclusions are as follows: satellite scale significantly influences the performance of hardening strategies. When the satellite scale is small, the MD strategy performs best; however, as the scale expands, the HO strategy surpasses MD and becomes the optimal choice. Across various satellite scales and removal strategies, the HO strategy maintains a consistently high AD value with low fluctuation. The scenario-adaptive rule is thus clarified: for small-scale satellite networks, the MD strategy is preferred to control costs, while for large-scale satellite networks, the HO strategy should be prioritized for optimal hardening effectiveness.

### 4.5. Vulnerability Analysis

**Figure 18:**
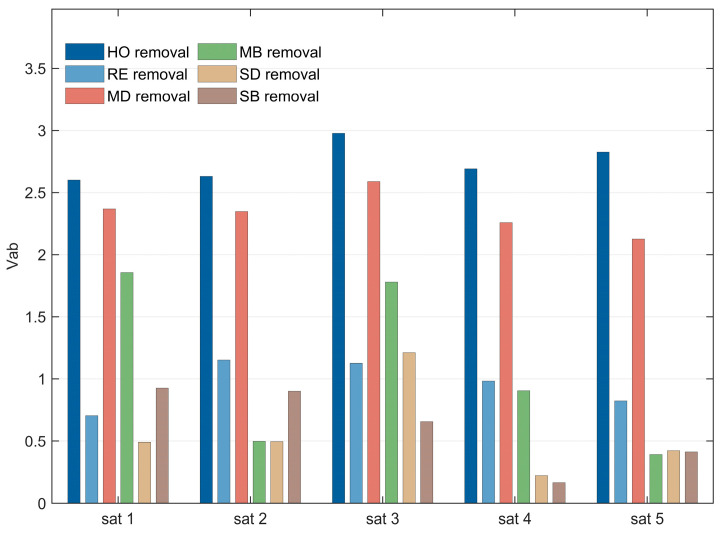
Vulnerability analysis of different satellite sizes.

**Figure 19:**
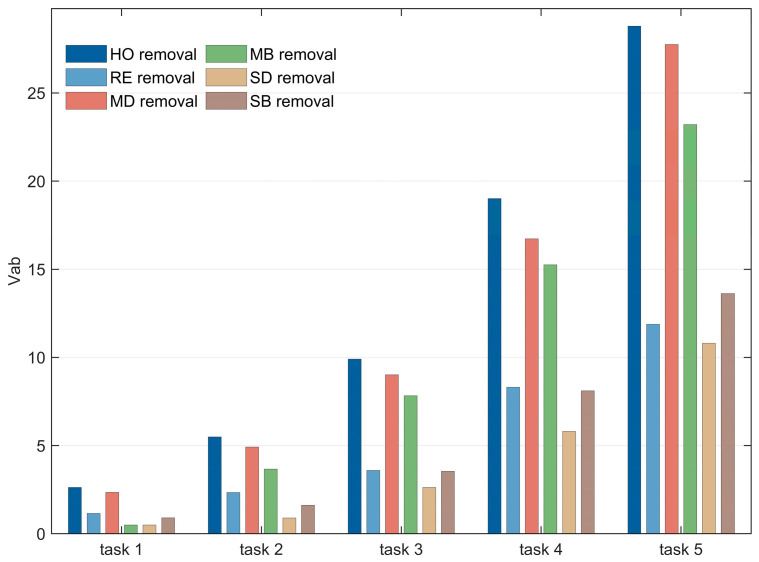
Vulnerability analysis at different task sizes.

This section combines Figure 18 and Figure 19 to analyze the variation in SIN vulnerability under different removal strategies from the two dimensions of satellite scale and mission scale. The correlation between network vulnerability (VAB) and these two scale factors is quantified, further verifying the reliability of the HO removal strategy in identifying key nodes and providing a basis for network protection and planning design. The vulnerability index VAB is defined as the difference between the initial network task efficiency and the task efficiency after damage. A larger VAB value indicates higher network vulnerability, meaning that node removal causes more severe degradation in network performance.

Figure 18 presents a comparison of network vulnerability under different satellite scales. The vertical axis represents the vulnerability index VAB (where a greater difference indicates higher vulnerability), and the removal step is fixed at 20. The results show that the VAB value of the HO removal strategy is the largest, significantly exceeding those of the other strategies, further validating the accuracy of its key node identification capability. The impact of satellite scale on network vulnerability is relatively minor, which can be attributed to the fixed mission scale in the experiment: satellite scale A already sufficiently meets mission requirements, and the addition of new satellites primarily increases link redundancy without substantially altering the importance of core nodes.

Figure 19 presents a comparison of network vulnerability under different task scales, with the removal step fixed at 20. The results indicate that task scale is positively correlated with network vulnerability: the larger the task scale, the higher the VAB value. Under larger task scales, the removal of core nodes causes more severe damage to the network due to the increased task contribution of these nodes. Furthermore, the performance gap between the HO strategy and other strategies widens as the task scale expands, highlighting its superiority in high task load scenarios.

In summary, through multiple sets of comparative experiments and detailed analysis of tabular data and graphical trends, this section comprehensively validates the effectiveness and adaptability of the proposed multi-layer hypernetwork model, HO removal strategy, and HO hardening strategy. The regulatory mechanisms by which satellite scale, mission scale, and orbit type influence mission efficiency, removal strategy performance, hardening effectiveness, and network vulnerability are systematically clarified. The advantages of the HO strategy in key node identification and network defense are demonstrated, providing solid experimental support and a theoretical foundation for subsequent validation in large-scale constellation scenarios and engineering applications.

## 5. Validation and Discussion Under Large-Scale Spatial Information Network Scenarios

This section primarily analyzes and discusses the task efficiency analysis, removal strategies, hardening strategies, and vulnerability of the proposed model in the context of large-scale SIN scenarios. The reliability and rationality of the model for large-scale constellations are validated, offering exploration directions for future research on SIN topology analysis.

### 5.1. Scenario Settings

To further verify the rationality and reliability of the multi-layer hypernetwork model, this section adopts a real large-scale constellation configuration, utilizing thousands of satellite nodes to construct an SIN analysis example. The reliability and rationality of the model are validated in large-scale constellation scenarios. The orbital parameters are consistent with those described in Section 2.1, while the constellation size parameters and mission scale settings are detailed in Table 8 and Table 9, respectively.

### 5.2. Mission Efficiency Analysis

**Figure 20:**
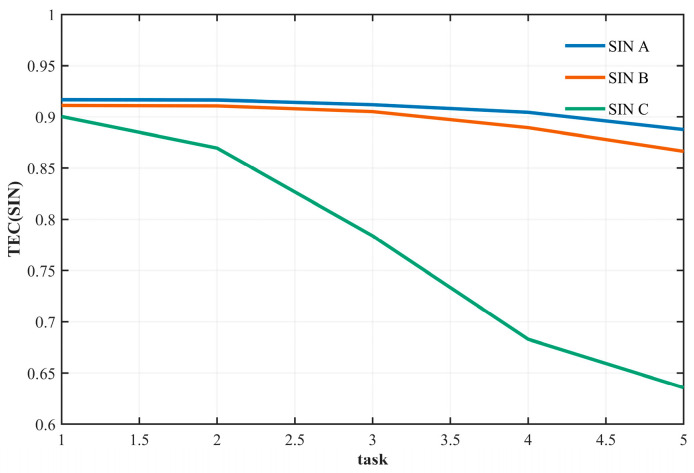
Large-scale constellation orbital type analysis.

Taking the large-scale constellations defined in Table 8 as an example, the impact of high, medium, and low Earth orbit satellites on mission efficiency is analyzed. The grouping experiments are designed as shown in Table 10:

As shown in Figure 20, the absence of high Earth orbit (HEO) satellites does not significantly affect the mission efficiency of the SIN, primarily because HEO satellites participate in relatively few missions. However, as the mission scale continues to increase, the efficiency gap gradually widens, indicating that HEO satellites become more involved in missions under larger task loads. Furthermore, when both MEO and HEO satellites are reduced to zero simultaneously, the mission efficiency difference remains small at low mission scales. Yet, as the mission scale grows, mission efficiency exhibits a noticeable decline. This suggests that at lower mission scales, missions are mainly supported by LEO satellites. As the mission scale expands, the participation of HEO satellites increases, and the contribution of MEO satellites to mission efficiency becomes particularly significant.

### 5.3. Removal Strategy Analysis

**Figure 21:**
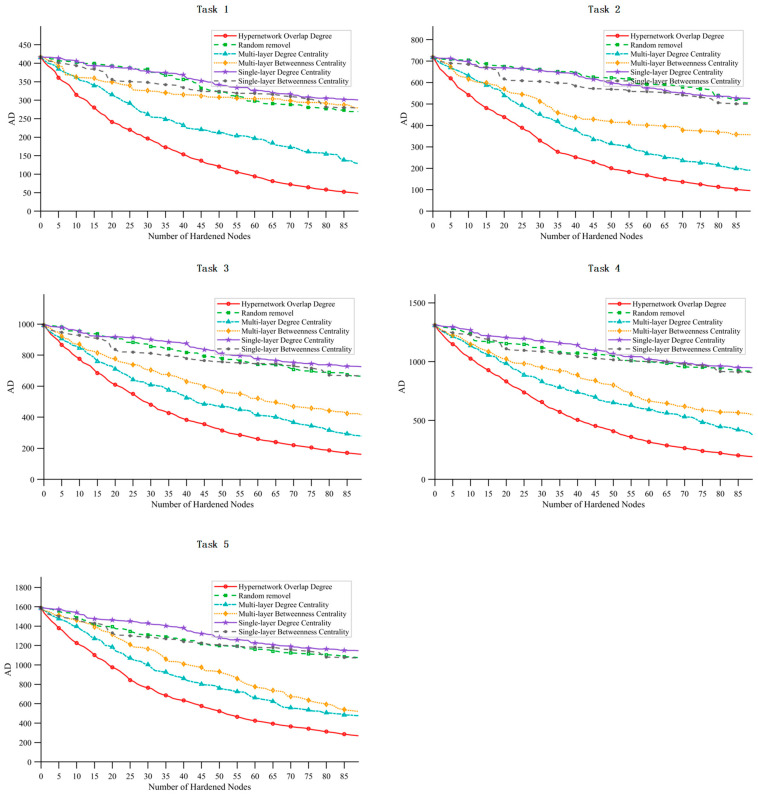
Remove Policy Comparison (Task Size).

Based on the comparative analysis in Section 4.2, this section focuses on the comparison of five typical removal strategies. As shown in Figure 21, the hypernetwork overlap (HO) removal strategy consistently performs well across different task scales. As the task scale continues to increase, the performance of large-scale SINs under multi-layer degree centrality (MD) and multi-layer betweenness centrality (MB) strategies gradually approaches that of HO, yet it always remains inferior. This finding is consistent with the conclusions drawn in Section 4.2.

### 5.4. Hardening Policy Analysis

**Figure 22:**
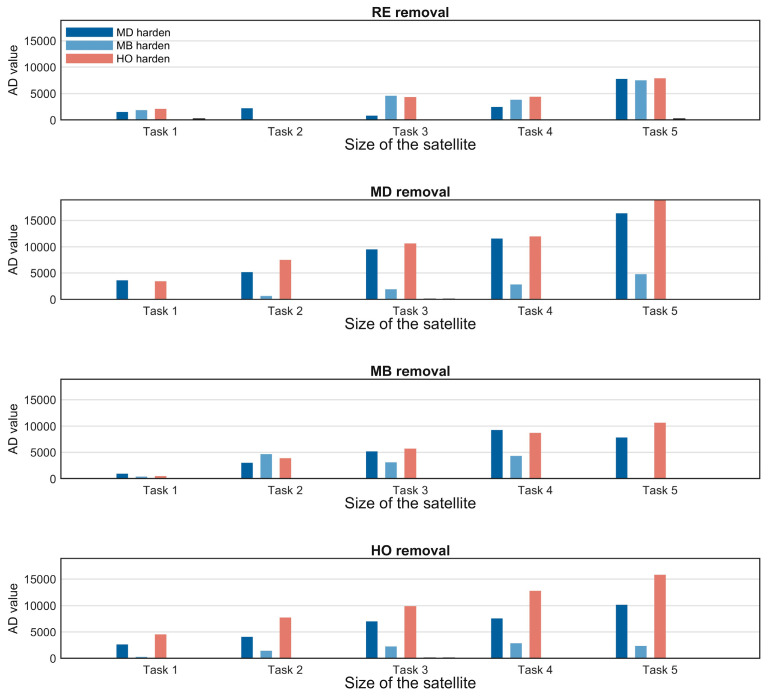
Hardening Policy Comparison.

This section focuses on large-scale SIN scenarios, conducting a performance analysis of node hardening strategies. Based on the preliminary analysis results from Section 4.3, the MD, MB, and HO hardening strategies significantly outperform other strategies. Therefore, these three strategies are selected to explore their performance characteristics under different task scales and different node removal strategies, as illustrated in Figure 22. The specific analysis is as follows:

1. Differences in Policy Effectiveness under Fixed Task Scale

Under a fixed task scale, the performance of the three hardening strategies is compared across different node removal strategies. A consistent pattern emerges: hardening efficiency is lowest when facing the RE (random removal) strategy and highest when facing the MD (multi-layer degree centrality-based) removal strategy. This result demonstrates the weakening effect of the uncertainty inherent in random removal on hardening effectiveness, while also validating the adaptive capability of hardening strategies against targeted removal methods.

2. Evolution of policy performance under different task sizes

Under different task scales, the performance differences among the three hardening strategies are analyzed for each removal strategy:

RE removal strategy scenario: Due to the randomness of the strategy, the performance differences among the three hardening strategies are not significant. As the task scale expands, their efficiency decline reduction (AD) tends to increase, which is a direct result of the larger area difference in AD calculation caused by the overall improvement in task efficiency.

MD removal strategy scenario: At small task scales, the performance of MD and HO is nearly equivalent, and both significantly outperform the MB hardening strategy. As the task scale expands, HO gradually surpasses MD in performance.

MB removal strategy scenario: At small task scales, the performance of the three hardening strategies tends to converge, with MB holding a slight advantage. This is attributed to the high alignment between the key nodes identified by the MB hardening strategy and the target nodes of the multi-layer betweenness centrality removal strategy. As the task scale increases, MD and HO significantly outperform MB, with HO exhibiting better performance under high task scales.

HO removal strategy scenario: From low to high task scales, the HO hardening strategy consistently outperforms the other two strategies.

In conclusion, the efficiency of the HO and MD hardening strategies consistently exceeds that of MB. Under low task scales or the RE removal scenario, MD performs best, which aligns with the preliminary conclusions of Section 4.3. However, as task scale expands, HO demonstrates superior efficiency across all types of removal strategies. From this, the adaptive scenario for hardening strategies in large-scale constellation networks can be inferred: MD is preferred under low task scale conditions, while HO should be chosen when task scale increases for optimal hardening effectiveness.

### 5.5. Vulnerability Analysis

Taking the large-scale constellation defined in Table 8 as an example, this section analyzes the vulnerability performance of SINs under different task scales when subjected to various removal strategies, both with and without the application of different hardening strategies. This analysis demonstrates the reliability of the proposed vulnerability assessment approach in real-world scenarios. On this basis, further analysis is conducted to examine the types of key nodes identified under different removal strategies.

1. Vulnerability Analysis

**Figure 23:**
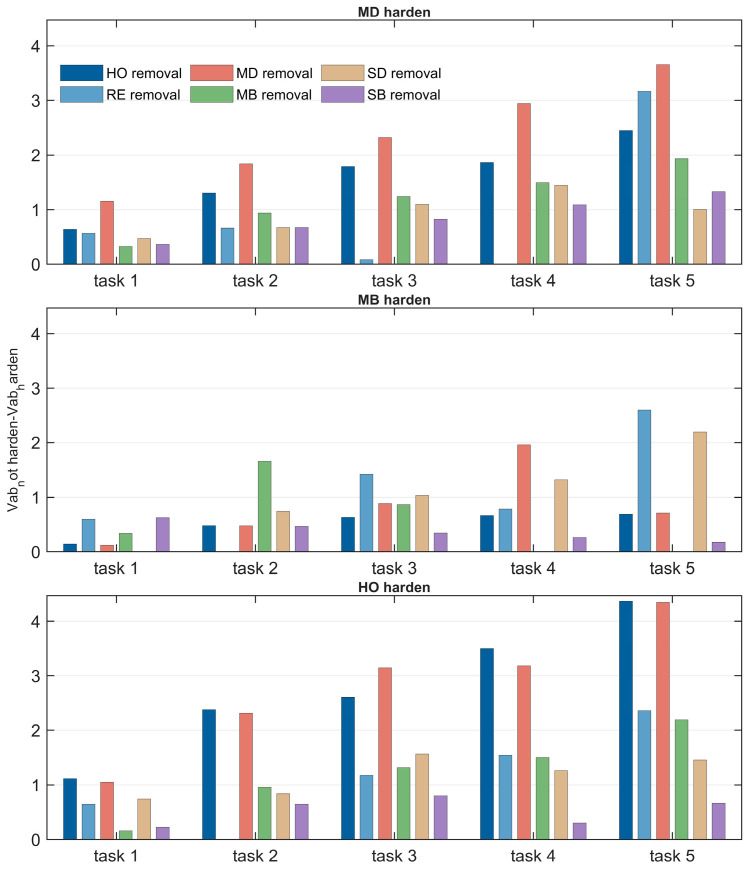
Hardening Strategy Analysis of Large-Scale Constellations.

According to the analysis in Section 4.4, SINs based on the large-scale constellations defined in Table 8 exhibit better performance under the three hardening strategies—MD, MB, and HO. Therefore, this section applies these three hardening strategies to reinforce the SIN, and subsequently evaluates network vulnerability under six removal strategies—HO, MD, MB, RE, SD, and SB. The vulnerability difference before and after hardening is calculated to assess the performance of each hardening strategy against different removal strategies. The results are presented in Figure 23.

It is observed that as task scale increases, the hardening effect becomes significantly more pronounced. This is because, with larger task scales, the number of tasks concurrently handled by each key node increases. Consequently, hardening these critical nodes substantially reduces the overall vulnerability of the network.

For the MD and MB hardening strategies, their protective effects are most pronounced when facing their corresponding removal strategies (i.e., MD and MB, respectively). However, the HO hardening strategy demonstrates superior hardening performance against the three most effective removal strategies—HO, MD, and MB—achieving a greater reduction in vulnerability compared to the other two hardening strategies. Moreover, the HO strategy also performs relatively well in mitigating the effects of the three less effective removal strategies. This sufficiently demonstrates that the HO hardening strategy can effectively reduce the vulnerability of SINs.

2. Hardening Node Type Analysis

**Figure 24:**
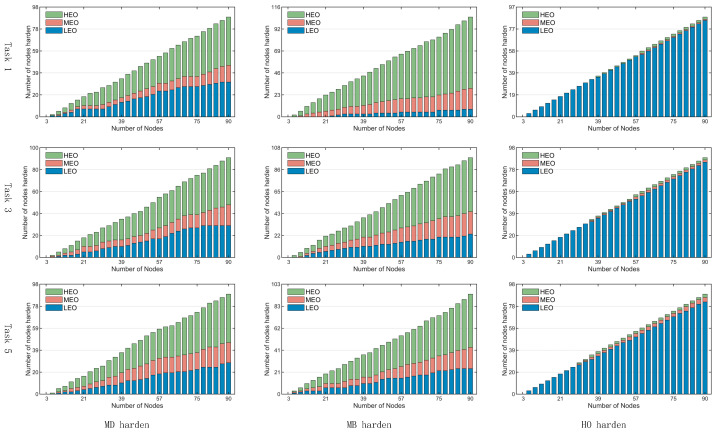
Hardening Node Type Analysis.

Further analysis is conducted on the three hardening strategies with superior performance—MD, MB, and HO—examining the types of hardened nodes selected under different task scales, as illustrated in Figure 24. From top to bottom, the subfigures correspond to task scales 1, 3, and 5; from left to right, they show the number of HEO, MEO, and LEO satellite nodes selected under the MD, MB, and HO hardening strategies, respectively.

The results reveal the following patterns:

Under the **MD hardening strategy**, the primary node types selected are HEO and LEO satellites. As the task scale increases, the number of MEO nodes selected also gradually increases.

Under the **MB hardening strategy**, the focus is predominantly on hardening HEO nodes. As task scale expands, the number of LEO nodes selected gradually increases.

Both the MD and MB strategies exhibit a common trend: they place greater emphasis on the connection relationships between nodes—that is, they prioritize physical links in the network topology. However, certain functions within SINs, such as navigation tasks, do not necessarily require direct physical links between nodes. In contrast, the **HO hardening strategy** is superior in that it fully accounts for the existence of navigation tasks and other mission requirements. Consequently, key nodes identified by HO are primarily concentrated among LEO satellites, and as the task scale increases, the proportion of HEO and LEO nodes selected continues to grow.

In conclusion, the multi-layer hypernetwork model proposed for SINs effectively enables vulnerability analysis, with both the HO removal strategy and the HO hardening strategy demonstrating superiority over other approaches. This fully illustrates that the hypernetwork model can more comprehensively extract characteristic indicators of the network topology. Through the analysis of hardened node types, valuable directions are provided for future efforts to reduce SIN vulnerability and to analyze regional vulnerability patterns. The findings indicate that particular attention should be paid to LEO-type nodes. Furthermore, by employing network characteristic analysis methods, important nodes exhibiting clustering characteristics can be identified, thereby enabling centralized key node mining for more effective network protection.

### 5.6. Discussion

The following aspects summarize the discussion on the reliability and effectiveness of the multi-layer hypernetwork model under large-scale constellation conditions:

First is the task efficiency analysis. The impact of orbit types on the task efficiency of SINs is scale-dependent: high-orbit satellites have a weak impact on task efficiency, while low-orbit satellites are the core of task execution. Among them, medium-orbit satellites contribute significantly to efficiency improvement as the task scale expands.

Second is the removal strategy analysis. The hypernetwork overlap (HO) removal strategy exhibits optimal performance under different task scales, significantly outperforming multi-layer degree centrality, multi-layer betweenness centrality, and other strategies. This validates the superiority of the hypernetwork model in mining network topology features.

Third is the hardening strategy analysis. This explains the performance patterns of task scale adaptation: the MD hardening strategy performs better in low task scale scenarios; the HO hardening strategy completely surpasses other strategies in performance after the task scale increases; and the HO strategy is most suitable against efficient removal strategies (MD, MB, HO), significantly reducing network vulnerability. On the other hand, it reveals essential differences in key node selection among different hardening strategies: the MD/MB strategy focuses on the physical connectivity characteristics of HEO and LEO nodes, while the HO strategy, by accommodating functional requirements such as navigation, concentrates core hardening nodes on LEO and increases the proportion of HEO nodes as the task scale expands.

In conclusion, the aforementioned multi-layer hypernetwork model can effectively support the vulnerability analysis of SINs, providing clear guidance for large-scale constellation instances: it is necessary to focus on LEO nodes to achieve centralized key node mining through clustering feature analysis, thereby improving network resilience.

## 6. Conclusions

With the scalable deployment of SINs and the expansion of application scenarios, enhancing their resistance to intentional attacks and malicious interference has become a core requirement for ensuring reliable network operation. Accurate and systematic vulnerability analysis has emerged as a leading research hotspot in the field of SIN topology optimization. This paper focuses on the vulnerability analysis of SINs by constructing a multi-layer hypernetwork model to characterize the vulnerability of network topologies under various node removal scenarios (including intentional attacks and interference). The effectiveness of the proposed removal and hardening strategies is validated through multiple sets of simulation experiments. The relevant conclusions provide theoretical and methodological guidance for SIN vulnerability analysis, specifically as follows:

First, a multi-layer hypernetwork model and a hypernetwork overlap removal strategy are proposed to adapt to three typical services—remote sensing, communication, and navigation—and accurately identify key nodes that participate in multiple high-value tasks simultaneously. When the satellite scale is large, this strategy exhibits a significant advantage, though its discriminative ability gradually decreases as the task scale expands.

Second, the effectiveness of the HO hardening strategy exhibits clear scale dependence. When the satellite scale is small, its advantage is not pronounced; however, its effectiveness improves significantly as the satellite scale expands. The strategy remains stable under various node attack scenarios, including both random and targeted removal, and can effectively reduce network vulnerability.

Third, the regulatory mechanisms of satellite scale, mission scale, and orbital hierarchy ratio on mission efficiency are clarified: the expansion of satellite scale has a positive gain on mission efficiency, while the expansion of mission scale exerts a nonlinear negative impact on the mission completion rate. The low Earth orbit (LEO) satellite constellation is identified as the node cluster requiring prioritized protection in SINs, and the mission contribution weights of satellites in different orbits are defined.

Fourth, a large-scale constellation is used as a topology prototype for example verification. The results demonstrate that the multi-layer hypernetwork analysis framework and the HO strategy proposed in this paper possess good applicability and effectiveness, accurately characterizing the vulnerability features of the network.

The practical application value of the above research results is of great significance for the engineering deployment of SINs. On one hand, the HO removal strategy can provide technical support for risk assessment under deliberate attacks in SINs, helping operation and maintenance personnel accurately locate critical weak nodes and predict potential losses caused by attacks in advance. On the other hand, the effectiveness of the HO hardening strategy can guide the allocation of node protection resources. When the satellite scale is small, a low-cost hardening strategy can be adopted; after scale expansion, the HO hardening strategy is preferred to achieve an optimal balance between defense cost and defense effectiveness. In addition, the regulatory mechanisms of satellite scale, mission scale, and orbit configuration can provide references for the planning and design of actual SINs, guiding the reasonable matching of construction scale with business requirements and avoiding resource waste. Focusing on the key protection of LEO satellite clusters can effectively improve the overall reliability and anti-interference capability of the network, ensuring the continuous and stable operation of core services such as remote sensing, communication, and navigation. The node characteristics and task contribution rules defined in this paper also lay a practical foundation for subsequent research on cost optimization in node removal and hardening strategies.

It should be noted that the vulnerability analysis framework for SINs proposed in this paper still has certain limitations. First, the model assumes that hardened nodes cannot be destroyed, which differs from real-world scenarios where hardened nodes may still be subjected to high-intensity interference and experience partial failure, thereby reducing the framework’s adaptability under extreme attack scenarios. Second, the analysis process does not account for the influence of differences in node hardware performance, link transmission loss, or external environmental factors such as space radiation and meteorological conditions. It assumes that node processing delay and link transmission delay are fixed values, which deviates from the dynamic characteristics of actual SINs. Third, the computational complexity of the HO strategy increases significantly as the satellite scale and task scale expand. In large-scale constellation scenarios involving tens of thousands of satellites or more, computational efficiency may become insufficient. Fourth, the framework does not consider scenarios involving multi-regional task coordination or dynamic switching of inter-satellite links, indicating that the versatility of the framework requires further improvement.

In view of the above limitations, future research will focus on the regional vulnerability of SINs and the technical challenges posed by large-scale deployment, with an emphasis on two major directions. First, the HO removal strategy algorithm will be optimized to meet the real-time and accurate analysis requirements of very-large-scale constellations. This involves reducing computational complexity and enhancing the capability for node mining that accounts for regional and task diversity. Second, practical factors such as node performance, environmental interference, and hardening costs will be incorporated, and multi-regional task coordination scenarios will be expanded to improve the versatility and practical adaptability of the framework. In addition, further study will be conducted on the efficient mining of regional key nodes and the dynamic optimization of network topology in response to service requirements.

## Figures and Tables

**Table 1:** Basic constellation parameters.

Type	Orbital Altitude (km)	Orbital Tilt (°)	Orbital Surface	Number of Satellites per Orbital Surface	Phase Factor
LEO	1200	55	10	10	1
MEO	20,000	55	5	5	1
HEO	35,786	55	1	3	1

**Table 2:** Task parameters.

Task Type	Communication	Navigation	Remote Sensing	
Node Type	Communication Node	Navigation node	Observation Node	Receive Node
Quantity Scale	50	50	10	5

**Table 3:** Zone parameters.

Region Type	Priority	Regional Latitude and Longitude		
Hotspot Area	3	[30,33; 118,123]	…	[49,52; −5,10]
General area	2	[32,38; 112,120]	…	[−39,−31; 140,150]
Unfollow areas	1	The rest of the region		

**Table 4:** Priority settings.

Node ID	Latitude	Longitude	Priority	Type
1	37.33	106.63	1	Communication
2	−222.56	−43.43	2	Communication
…	…	…	…	
55	−25.37	−49.45	1	Remote Sensing
56	31.07	128.82	1	Remote Sensing
…	…	…	…	
115	31.47	100.39	1	Navigation

**Table 5:** Plan Settings.

Group	C	R	N	T	LEO	O-L	MEO	O-M	HEO	O-H	S
1-1	50	10	50	110	100	10	25	5	3	1	128
1-2	50	10	50	110	121	11	36	6	4	2	161
1-3	50	10	50	110	144	12	49	7	9	3	202
1-4	50	10	50	110	169	13	64	8	16	4	249
1-5	50	10	50	110	196	14	81	9	25	5	302
2-1	100	20	100	220	100	10	25	5	3	1	128
…	…	…	…	…	…	…	…	…	…	…	…
3-1	200	40	200	440	100	10	25	5	3	1	128
…	…	…	…	…	…	…	…	…	…	…	…
4-1	300	60	300	660	100	10	25	5	3	1	128
…	…	…	…	…	…	…	…	…	…	…	…
5-1	400	80	400	880	100	10	25	5	3	1	128
…	…	…	…	…	…	…	…	…	…	…	…
5-5	400	80	400	880	196	14	81	9	25	5	302

**Table 6:** **The value of correlation coefficient**.

Correlation Coefficient	Value	Correlation Coefficient	Value
$t_{0}$	Dimensionless	$\sigma_{com}$	0.75
$\sigma_{rs}$	0.55	α	0.8

**Table 7:** Plan Settings.

Satellite Size	LEO Quantity	LEO Track Surface	MEO Quantity	MEO Track Surface	HEO Quantity	HEO Track Surface
1	100	10	25	5	3	1
2	144	12	25	5	3	1
3	196	14	25	5	3	1
4	256	16	25	5	3	1
5	324	18	25	5	3	1
6	100	10	36	6	3	1
7	100	10	49	7	3	1
8	100	10	64	8	3	1
9	100	10	81	9	3	1
10	100	10	25	5	6	2
11	100	10	25	5	9	3
12	100	10	25	5	12	4
13	100	10	25	5	15	5

**Table 8:** Constellation Size Parameters.

	LEO Quantity	LEO Track Surface	MEO Quantity	MEO Track Surface	HEO Quantity	HEO Track Surface
Parameters	648	18	324	18	81	9

**Table 9:** Task Size Parameters.

Task Type	Communication Task	Navigation Task	Remote Sensing Task	
Node Type	Communication Node	Navigation Node	Observation Node	Receiving Node
Task Size 1	300	300	30	15
Task Size 2	700	700	70	35
Task Size 3	1100	1100	110	55
Task Size 4	1500	1500	150	75
Task Size 5	1900	1900	190	95

**Table 10:** Plan Settings.

Group	LEO	MEO	LEO	Total
SIN A	648	324	81	1053
SIN B	648	324	0	972
SIN C	648	0	0	648

## Data Availability

The original contributions presented in the study are included in the article. Further inquiries can be directed to the corresponding author.

## Abbreviations

The following abbreviations are used in this manuscript:

SIN	Spatial Information Network
LEO	Low Earth Orbit
MEO	Medium Earth orbit
HEO	High Earth Orbit
RE	Random node removal strategy
SD	Single-layer Degree Centrality
SB	Single-layer Betweenness centrality
MD	Multilevel Degree Centrality
MB	Multilevel Betweenness centrality
HO	Hypernet overlap removal strategy (HO)

## Symbols

**Symbol**	**Description**	**Unit**
A	Scale of the hyperedge	–
AD	Area difference of efficiency curves (hardening effect indicator)	–
C	Number of communication tasks	–
CR	Task completion rate	–
E	Finite set of physical edges in the spatial information network	–
H	Set of task hyperedges	–
m	Number of visible LEO satellites at the navigation node	–
N	Number of navigation tasks	–
R	Number of remote sensing tasks	–
S	Total number of satellites in the constellation	–
$S_{v}$	Hypernetwork overlap importance score of node v	–
T	Total number of tasks	–
TE	Overall task efficiency of spatial information network	–
$TE_{comm}$	Communication task efficiency	–
$TE_{nav}$	Navigation task efficiency	–
$TE_{rs}$	Remote sensing task efficiency	–
V	Finite set of nodes in the spatial information network	–
$V_{ab}$	Vulnerability indicator of spatial information network	–
v	Node in the hypernetwork	–
t	Shortest delay from the task hyperedge mid-source node to the target node	s
$a_{comm}$	Communication transmission coefficient	–
$a_{nav}$	Navigation coefficient	–
$a_{rs}$	Remote sensing transmission coefficient	–
$\eta_{i}$	Efficiency of the i-th task	–
$\tau_{e}$	Transmission latency of the edge	s
$\tau_{n}$	Forwarding latency of the node	s
$\omega_{i}$	Priority weight of the i-th task	–
